# A comparative study of forest methods for time-to-event data: variable selection and predictive performance

**DOI:** 10.1186/s12874-021-01386-8

**Published:** 2021-09-25

**Authors:** Yingxin Liu, Shiyu Zhou, Hongxia Wei, Shengli An

**Affiliations:** Department of Biostatistics, School of Public Health (Guangdong Provincial Key Laboratory of Tropical Disease Research), Southern Medical University, Guangzhou, Guangdong China

**Keywords:** Survival analysis, Random survival Forest, Conditional inference Forest, Maximally selected rank statistics, Machine learning, Variable selection, Brier score

## Abstract

**Background:**

As a hot method in machine learning field, the forests approach is an attractive alternative approach to Cox model. Random survival forests (RSF) methodology is the most popular survival forests method, whereas its drawbacks exist such as a selection bias towards covariates with many possible split points. Conditional inference forests (CIF) methodology is known to reduce the selection bias via a two-step split procedure implementing hypothesis tests as it separates the variable selection and splitting, but its computation costs too much time. Random forests with maximally selected rank statistics (MSR-RF) methodology proposed recently seems to be a great improvement on RSF and CIF.

**Methods:**

In this paper we used simulation study and real data application to compare prediction performances and variable selection performances among three survival forests methods, including RSF, CIF and MSR-RF. To evaluate the performance of variable selection, we combined all simulations to calculate the frequency of ranking top of the variable importance measures of the correct variables, where higher frequency means better selection ability. We used Integrated Brier Score (*IBS*) and c-index to measure the prediction accuracy of all three methods. The smaller *IBS* value, the greater the prediction.

**Results:**

Simulations show that three forests methods differ slightly in prediction performance. MSR-RF and RSF might perform better than CIF when there are only continuous or binary variables in the datasets.

For variable selection performance,

When there are multiple categorical variables in the datasets, the selection frequency of RSF seems to be lowest in most cases. MSR-RF and CIF have higher selection rates, and CIF perform well especially with the interaction term.

The fact that correlation degree of the variables has little effect on the selection frequency indicates that three forest methods can handle data with correlation.

When there are only continuous variables in the datasets, MSR-RF perform better. When there are only binary variables in the datasets, RSF and MSR-RF have more advantages than CIF.

When the variable dimension increases, MSR-RF and RSF seem to be more robustthan CIF

**Conclusions:**

All three methods show advantages in prediction performances and variable selection performances under different situations. The recent proposed methodology MSR-RF possess practical value and is well worth popularizing. It is important to identify the appropriate method in real use according to the research aim and the nature of covariates.

**Supplementary Information:**

The online version contains supplementary material available at 10.1186/s12874-021-01386-8.

## Background

Survival analysis, also known as time-to-event analysis, is a branch of statistics investigated in how long it takes for certain events to occur, and estimating the relevant important factors. A key feature of these time-to-event datasets is that they contain either censored or truncated observations, in which right censoring is the most commonly encountered type [1]. The Cox-proportional hazards regression model (Cox model) is a default choice in analyzing right-censored time-to event data [2]. As a semi-parametric method, its flexibility derives from requiring no specifications of the shape of the hazard function, which means no assumption is required on the overall shape of survival times [3]. However, its restrictive proportional hazards assumption is always not met in applications [4–6]; what’s more, the covariates are assumed to have an additive effect on the log hazard ratio, which may become unsuitable for data containing non-linearity or high dimensional covariates [7, 8]. Machine learning methods can deal with these data. Machine learning methods have been widely concerned in the biomedical field because of their great abilities for self-studying, classification, prediction and feature identification, among which the forests approach is especially popular with scholars and researchers.

The random forests (RF) approach was first proposed by Breiman [9]. RF construct ensembles from tree base learners, and then combine the results to a final decision. In RF, randomness is introduced in two forms: First, each of the randomly drawn bootstrap samples of the data is used to grow a tree [10]. Second, at each node of the tree, a randomly selected subset of covariates is chosen as candidate variables for splitting [11]. With CART being base learner, the original RF primarily focus on classification and regression problems [12]. Random survival forests (RSF) methodology proposed by Ishwaran et al. extends RF method to right-censored time-to-event data [13, 14]. RSF can easily handle high dimensional covariate data as RF [15–17]. However, RSF also inherit the drawbacks of RF, especially the selection bias towards covariates with more possible split points, which may result in bias of other parameter estimates such as variable importance measures [18].

Conditional inference forests (CIF) methodology is known to reduce selection bias via a two-step split procedure implementing hypothesis tests [19]. Instead of maximizing a splitting criterion over all possible splits simultaneously in RSF, CIF separate the algorithms for the best split variable search and the best split point search [20]. In the first step, a linear rank association test is performed to determine the optimal split variable. In the second step, the optimal split point is determined by comparing two-sample linear statistics for all possible partitions for the split variable. Despite the two steps are both implemented within the theory of permutation tests, there is a change in the statistical approach for the split variable and the split point selection, which increases the time and storage of CIF application.

Random forests with maximally selected rank statistics (MSR-RF) methodology proposed by Wright et al. seems to be a great improvement towards RSF and CIF [21]. Following the basic concept of CIF, MSR-RF use a two-step split procedure via hypothesis tests, which means MSR-RF also separate the variable selection and the split point procedures. However, distinguished from CIF, binary split via maximal log rank score is used consistently in both steps of MSR-RF, which saves time and reduces bias. Log rank score is one of the most commonly used criterion statistics in RSF. What’s more, the authors Wright et al. introduced a new package ***ranger*** proved to be faster [22]. This package can be used in both C and R languages, which makes MSR-RF more feasible.

Despite the development of survival forests, only a few studies have been done to compare the forests methods. MSR-RF’s authors did simulations to illustrate their methods with RSF and CIF as reference, including split variable selection performance for the null case of no association between covariates and survival outcome, prediction performance under several situations, and runtime performance [21]; Nasejje et al. did simulation study to compare the prediction performance between RSF and CIF with all variables associated with the survival outcome, while split variable selection performance was not investigated [23]; Du et al. compared the prediction performance between RSF and CIF on real cancer dataset without split variable selection performance [24]. The previous simulation researches majorly focused on the predictive performance of the methods without considering the variable selection performance. What’s more, proposed in 2016, MSR-RF methodology still has not been implemented in those recent researches, while RSF and CIF retain the wide use. The main aim of this research is popularize the MSR-RF methodology and to provide advices of using the survival forests methods concerning on variable selection and prediction. We think it’s essential to study in depth and compare the survival forests under different situations. In this paper we used simulation study and real data study to compare prediction performances and variable selection performances among three survival forests mentioned above, including RSF, CIF and MSR-RF.

The article is structured as follows: section 2 “Methods” describes the three methods used. In section 3 “Simulation study”, we present the simulation study together with the simulation results. Section 4 “Application study” introduces the two real datasets used in this study and also gives the corresponding real data analysis results. Lastly section 5 “Discussion and conclusion” presents the discussion and conclusions drawn from this study.

## Methods

### Random survival forests

Random survival forests (RSF) method is an extension of Brieman’s RF method to right censored time-to-event data [13]. Given the original data with *N* subjects and *M* features, RSF algorithm is described as follows:

1. Draw *B* bootstrap samples from the original data. Bagging generates *B* new training sets with replacement [10]. If the size of each training set equals to *N*, each subject in the original data has a probability of (*1–1*/*N*)^*N*^ not being selected. In this way, on average 36.8% of the data would be excluded for each bootstrap sample, called out-of-bag data (OOB data) [9].
2. Grow a binary survival tree for each bootstrapped sample. At each node of the tree, randomly select *m* (*m* < < *M*) features for splitting. In practical settings *m* is usually set to *m=* $\sqrt{M}$ or *m = log*_*2*_*M*. A split is made using the candidate feature and its cut-off point that maximizes the survival differences between daughter nodes under a predetermined split rule [14].
3. Grow the tree to full size under the pre-specified constraints.
4. Calculate a cumulate hazard function (CHF) and a survival function (SF) for each tree. Average over all trees to obtain the ensemble CHF. In this way, one estimate for each individual in the data is calculated.
5. Using OOB data, calculate prediction error for the ensemble CHF and variable importance measures (*VIM*) of *M* features.

Researchers have come up with several splitting rules for RSF, among which four rules are representative [13], including: a log-rank splitting rule that splits nodes by maximization of the log-rank test statistic, a log-rank score splitting rule that splits nodes by maximization of a standardized log-rank score statistic, a conservation-of-events splitting rule that splits nodes by finding daughters closest to the conservation-of-events principle, a random log-rank splitting rule that splits nodes by the variable with maximum log-rank statistic (at its predetermined random split point). Log-rank splitting rule and log-rank score splitting rule are the most popular rules in practical use. Log-rank splitting rule is described as follows:

The log-rank test for a parent node splitting at the cut-off point value *c* for predictor *X*_*j*_ is

$$L\left({X}_j,c\right)=\frac{\sum_{k=1}^K\left({d}_{k,l}-{Y}_{k,l}\frac{d_k}{Y_k}\right)}{\sqrt{\sum_{k=1}^K\frac{Y_{k,l}}{Y_k}\left(1-\frac{Y_{k,l}}{Y_k}\right)\left(\frac{Y_k-{d}_k}{Y_k-1}\right){d}_k}}$$

Let *t*_1_ < *t*_2_ < … < *t*_*K*_ be the distinct death times in the parent node, *d*_*k*_ and *Y*_*k*_ equal the number of deaths and individuals at risk at time *t*_*k*_ in the parent node respectively. *Y*_*k*_ = *Y*_*k*, *l*_ + *Y*_*k*, *r*_, *d*_*k*_ = *d*_*k*, *l*_ + *d*_*k*, *r*_. *d*_*k*, *l*_ and *Y*_*k*, *l*_ represent those in the left daughter node, which means *Y*_*k*, *l*_ = {*i* : *t*_*i*_ ≥ *t*_*k*_, *X*_*ji*_ ≤ *c*}. The value of |*L*(*X*_*j*_, *c*)| is the measure of node separation. The larger the value of |*L*(*X*_*j*_, *c*)|, the greater the survival difference between the two groups. The best split is determined by finding the predictor *X*_*j*∗_ and split value c* with maximum statistic value.

RSF naturally inherit many of RF’s good properties [16], including: non-parametric, flexible, and can easily handle high dimensional covariate data, which are essential in the genetics field; RSF are highly data adaptive and model assumption free, which are especially helpful when associations between predictors and outcome are complex such as nonlinear effects or high-order interactions; what’s more, *VIM* and OOB estimates can be obtained through the forest growing. RSF can be performed through several packages. Here we use R package ***randomForestSRC*** [25]. Log-rank splitting rule is implemented.

### Conditional inference forests

Conditional inference forests (CIF) method is a tree ensemble method utilizing the theory of permutation tests [19, 26]. As CART serves as base learner in RF, this kind of algorithms has a variable selection bias towards variables with many split points. This bias is induced by maximizing a splitting criterion over all possible splits, whereas the chance to find a good split increases if the variable has more split points. The authors thought even an uninformative variable could also sit high up on the tree’s structure, and then result in biased estimate [18]. CIF are known to solve this problem by taking statistical significance into account [27].

CIF construct forests with conditional inference tree (CIT) as base learner [19]. Instead of maximizing a splitting criterion over all possible splits, CIT separates the algorithms for selecting the best split covariate from the best split point search. CIT first conducts association tests to determine the best split covariate, and then makes the best binary split based on standardized linear statistic.

Same as RSF, assume the original data with *N* subjects and *M* features, and a new training set is defined as ${\mathcal{L}}_n=\left\{\left({Y}_i,{X}_{1i},\dots, {X}_{Mi}\right);i=1,\dots, n\right\}$. At step 1 variable selection of CIT splitting procedure, we need to decide whether there is any information about the response variable covered by covariate *X*_*j*_, which is indicated by partial hypothesis of independence ${H}_0^j:D\left(Y|{X}_j\right)=D(Y)$ with global null hypothesis ${H}_0=\bigcap_{j=1}^M{H}_0^j$. The association between Y and *X*_*j*_, is measured by linear statistics of the form

$${T}_j\left({\mathcal{L}}_n,w\right)= vec\left({\sum}_{i=1}^n{w}_i{g}_j\left({X}_{ji}\right)h{\left({Y}_i,\left({Y}_1,\dots, {Y}_n\ \right)\right)}^{\intercal}\right)$$

Where *w* is case weight indicating each node, *g*_*j*_ is a non-random transformation of the covariate *X*_*j*_, The influence function *h* depends on the responses (*Y*_1_, …, *Y*_*n*_) in a symmetric permutation way. These functions may differ in practical settings, such as in time-to-event data the influence function may be chosen as log rank score or Savage score. The evaluation of ${T}_j\left({\mathcal{L}}_n,w\right)$ is based on the distribution of Y and *X*_*j*_, which often remains unknown. However, at least under the null hypothesis one can dispose of this dependency by fixing the covariates and conditioning on all possible permutations of the responses, which is known as the theory of permutation tests. Later in the algorithm, ${T}_j\left({\mathcal{L}}_n,w\right)$ is standardized to univariate test statistics $u\left|{T}_j\left({\mathcal{L}}_n,w\right)\right|$ for further comparison. If we are not able to reject *H*_0_ at a pre-specified level α, we stop the recursion, otherwise select *X*_*j*∗_ with the strongest association (the smallest *P* value) as the best split variable.

Once we have selected a covariate *X*_*j*∗_ at step 1 of the algorithm, an optimal split point should be determined at step 2. The goodness of a split is evaluated by a two-sample linear statistics which is a special case of the linear statistic used at step 1. For all possible split points of *X*_*j*∗_ the linear statistic is

$${T}_{j\ast}^c\left({\mathcal{L}}_n,w\right)= vec\left({\sum}_{i=1}^n{w}_i\mathrm{I}\left({X}_{j\ast i}\le c\right)h{\left({Y}_i,\left({Y}_1,\dots, {Y}_n\ \right)\right)}^{\intercal}\right)$$

The two-sample statistic measures the discrepancy between two daughter nodes. The split *c∗* with a standard test statistic $u\left|{T}_{j\ast}^c\left({\mathcal{L}}_n,w\right)\right|$ maximized over all possible splits is established.

CIF differ from RF and RSF with respect to not only base learner but the aggregation scheme applied. Instead of averaging predictions directly as in RF, the aggregation scheme works by averaging observation weights extracted from each tree. CIF are implemented in the R package called ***party*** [28].

### Survival forests with maximally selected rank statistics

Survival forests with maximally selected rank statistics (MSR-RF) method was proposed by Wright et al. in 2017 [21]. The authors thought an obvious disadvantage of standard CIF was a change in the statistical approach for split variable and split point selection. As is introduced above, the association test for selecting the split variable is based on a linear rank statistic, while the optimal split is a dichotomous threshold-based split. MSR-RF are designed to deal with those problems by a statistical test for binary splits using maximally selected rank statistics [29].

The forest algorithm of MSR-RF is identical with that of RSF. Randomness is induced in both selections of samples and covariate subsets. Finally results of the trees are aggregated through vote or average. The split procedure of MSR-RF follows the basic concept of CIF, which means a two-step procedure via hypothesis tests separating variable selection and split point search. Maximally selected rank statistics for survival endpoints is implemented through log-rank score, which also can be used in RSF and CIF as we mentioned above.

Consider a training set ${\mathcal{L}}_n=\left\{\left({Y}_i,{X}_{1i},\dots, {X}_{Mi}\right);i=1,\dots, n\right\}$ at a node, for time-to-event data *Y*_*i*_ = (*t*_*i*_, *δ*_*i*_), where *t*_*i*_ is survival time and *δ*_*i*_ is censoring indicator. To describe the log-rank score splitting rule, assume the covariate *X*_*j*_ has been ordered so that *X*_*j*1_ ≤ *X*_*j*2_ ≤ … ≤ *X*_*jn*_. The log-rank score is defined as a “rank” for each survival time *t*_*i*_

$${a}_i={\delta}_i-{\sum}_{k=1}^{\Gamma_i}\frac{\delta_k}{n-{\Gamma}_k+1}$$

Where Γ_*i*_ is the number of observations with survival time up to *t*_*i*_. The linear rank statistics for a split at point *c* is the sum of all log-rank scores in the left daughter node ${\sum}_{X_{ji}\le c}{a}_i$. The null hypothesis is ${H}_0^j:P\left(Y|{X}_j\le c\right)=P\left(Y|{X}_j>c\right)$ for all points. Under the null hypothesis, the standardized log-rank score test statistic is

$$S\left({X}_j,c\right)=\frac{\sum_{X_{ji}\le c}{a}_i-{n}_l\overline{a}}{\sqrt{\frac{n_l{n}_r{S}_a^2}{n}}}$$

Where $\overline{a}$ and ${S}_a^2$ are the sample mean and sample variance of *a*_*i*_, *n*_*l*_ = {*i* : *X*_*ji*_ ≤ *c*} denotes the number in the left daughter node, *n* = *n*_*l*_ + *n*_*r*_. Log-rank score splitting defines the measure of node separation by |*S*(*X*_*j*_, *c*)|. The maximum statistic value yields the best split, and is defined as the maximally selected rank statistics (MSR).

In RSF, a split is established by maximizing a splitting criterion over all possible splits, where the values of log-rank score test statistics would be compared not only between cut-points on the same variable but also between different variables, which induces bias. MSR-RF deal with the problem with a two-step procedure. In the first step, for each potential variable, the split point with the maximally selected rank statistics is selected. Therefore, for each variable, *P* values are obtained for the best split point under the null hypothesis. The covariate with the smallest *P*-value is selected as splitting candidate. Only if the adjusted *P*-value for multiple testing of the candidate is smaller than the pre-specified type I error, the split is made, otherwise no split is performed. In the second step, MSR-RF procedure simplifies CIF procedure as the optimal split point is determined as a by-product in step 1, which means new computation is needed no more in step 2. In this way, one procedure is used consistently in both steps of MSR-RF.

MSR-RF model is implemented in the R package called ***ranger*** [30].

## Simulation study

### Simulation design

In this section, we conducted simulation study to evaluate the performance of the three survival forests described in the previous section, in terms of prediction and variable selection.

The number of Monte Carlo simulation replications was set to 1000. All forests were run with 200 trees, in which the number of candidate covariates *m* for splitting was set to square root of the number of covariates *M*. The significance level of all hypothesis tests in this study was set to 0.05. To avoid the problems of overfitting that arises from using the same dataset to train and test model, in each simulation we randomly selected 80% subjects as training set and the other 20% as test set.

Survival time *T* was generated by inverting survival function via exponential distribution.

$$T=-\mathit{\log}(U)\ast \exp \left(0.5+{\beta}^T{X}_i\right)$$

Where *U* followed the uniform distribution *U*(0, 1). Censoring times were generated from exponential distributions with different parameters to get different censoring rates.

The models that generated datasets are listed in Table 1, where the form is described as *β*^*T*^*X*_*i*_. For each simulated dataset, only two covariates were set to be associated with survival outcome, whereas the others performed as noise covariates. We specified four types of models:

A. Multiple categorical covariates are included, and no interaction term exists.
B. Multiple categorical covariates are included, and one first-order interaction term exists.
C. Only continuous covariates generated from multivariate normal distribution are included, and the correlation degree among covariates changes.
D. Only independent and identical distributed covariates are included, and the dimension of covariates changes.

**Table 1:** Information of each simulated dataset

model	form	covariate
A1	1.5*x*_1*i*_ + Ι(*x*_5*i*_ = 2)	*x*_1*i*_ − *x*_2*i*_~U(0, 1), *x*_3*i*_ − *x*_4*i*_~Ν(0, 1), *x*_5*i*_ − *x*_6*i*_~*Discrete*U(1, 2), *x*_7*i*_ − *x*_8*i*_~*Discrete*U(1, 4), *x*_9*i*_ − *x*_10*i*_~*Discrete*U(1, 8)^*a*^
A2	1.5*x*_1*i*_ + Ι(*x*_7*i*_ ≥ 3)	
A3	1.5*x*_1*i*_ + Ι(*x*_9*i*_ ≥ 5)	
B1	Ι(*x*_1*i*_ > 0.5) ∗ Ι(*x*_5*i*_ = 2)	
B2	Ι(*x*_1*i*_ > 0.5) ∗ Ι(*x*_7*i*_ ≥ 3)	
B3	Ι(*x*_1*i*_ > 0.5) ∗ Ι(*x*_9*i*_ ≥ 5)	
C	*x*_1*i*_ + 1.5*x*_2*i*_	*x*_1*i*_ − *x*_10*i*_~*MV*Ν(0, Σ)^*b*^
D1	2*x*_1*i*_ + 3*x*_2*i*_	*x*_1*i*_ − *x*_*Mi*_~Ν(0, 1)^c^
D2	2*x*_1*i*_ + 3*x*_2*i*_	*x*_1*i*_ − *x*_*Mi*_~*Discrete*U(1, 2)^a^

^a^ *Discrete*U(1, *k*) is the discrete uniform distribution, a simple distribution that puts equal weight on the integers from 1 to k

^b^ Σ is a squared matrix with all diagonal elements equal to 1 and all off-diagonal elements equal to ρ

^c^*M* is the number of covariates

Model A was established in a linear form with ten covariates, including four continuous covariates *x*_1*i*_ − *x*_4*i*_ (two covariates were generated from uniform distribution *x*_1*i*_ − *x*_2*i*_~U(0, 1) and the others were generated from standard normal distribution *x*_3*i*_ − *x*_4*i*_~Ν(0, 1)) and six categorical covariates *x*_5*i*_ − *x*_10*i*_ (the categorical covariates were generated from discrete uniform distributions with different categories, including two covariates with 2 categories *x*_5*i*_ − *x*_6*i*_~*Discrete*U(1, 2), two covariates with 4 categories *x*_7*i*_ − *x*_8*i*_~*Discrete*U(1, 4) and two covariates with 8 categories *x*_9*i*_ − *x*_10*i*_~*Discrete*U(1, 8)). Only a continuous covariate *x*_1*i*_ and an unordered-categorical covariate were set to be associated with the outcome, including (A1) covariate *x*_5*i*_ with 2 categories; (A2) covariate *x*_7*i*_ with 4 categories; (A3) covariate *x*_9*i*_ with 8 categories. For valid comparison, we controlled the categories in the indicative function *Ι*() so that 50% subjects in each model would have a value of 1. Model A was simulated at different censoring rates of 0, 25, 50, 75% and different sample sizes of 100, 200, 400, 800 (training set).

Model B had the same covariate framework as model A. Model B was established in a first-order interaction form with a continuous covariate *x*_1*i*_ and an unordered-categorical covariate associated with the outcome, including (B1) covariate *x*_5*i*_ with 2 categories; (B2) covariate *x*_7*i*_ with 4 categories; (B3) covariate *x*_9*i*_ with 8 categories. Model B was simulated at different censoring rates of 0, 25, 50, 75% and different sample sizes of 100, 200, 400, 800 (training set).

Model C was established in a linear form with two continuous covariates *x*_1*i*_ and *x*_2*i*_ associated with the outcome. In this model all ten variables followed multivariate normal distribution *MV*Ν(0, Σ), where Σ is a squared matrix with all diagonal elements equal to 1 and all off-diagonal elements equal to ρ. We changed the parameter ρ to get different correlations between the covariates. Model C was simulated at different censoring rates of 0, 25, 50, 75%, different sample sizes of 40, 100, 200, 400, 800 (training set) and different correlation parameter ρ of 0, 0.2, 0.4, 0.6, 0.8.

Model D was established in a linear form with two covariates *x*_1*i*_ and *x*_2*i*_. It was used to study the performance of the methods under different dimensions of covariates, including (D1) continuous covariates all generated from the standard normal distribution; (D2) binary covariates all generated from discrete uniform distribution. The sample size *N* was set to 100 (training set); the censoring rate was set to 0, 25, 50, 75%. The ratio *M/N*, which means the ratio of the number of covariates *M* to the sample size *N*, was set to 0.2, 0.5, 1, 2, 5.

### Model evaluation

To evaluate the performance of variable selection, we ranked the *VIM* of each forest in each simulation and obtained the ranks of the two correct variables. Finally we combined all simulations to calculate the frequency of the correct variables ranking in the top by *VIM.*

We used prediction error based on Brier score and c-index (c-index only exhibited in the supplement) to measure the prediction accuracy of all the three models. Brier score was originally applicable to multi-category forecasts, defined as the mean squared difference between the predicted probabilities and the actual observations [31].

$$BS=\frac{1}{N}\sum_{i=1}^N\sum_{j=1}^R{\left({predict}_{ij}-{observe}_{ij}\right)}^2$$

Where *N* is sample size and *R* is the number of categories, *predict*_*ij*_ is the predicted probability for individual *i* assigned to the possible category *j*, and *observe*_*ij*_ is the actual observation for individual *i* at category *j* (1 if it is the actual observation and 0 otherwise).

The brier score *BS*(*t*) for survival data is defined as a function of time

$$BS(t)=\frac{1}{N}\sum_{i=1}^N\left\{{\left[0-\hat{S}\left(t|{X}_i\right)\right]}^2\frac{I\left({t}_i\le t,{\delta}_i=1\right)}{\hat{G}\left({t}_i|{X}_i\right)}+{\left[1-\hat{S}\left(t|{X}_i\right)\right]}^2\frac{I\left({t}_i>t\right)}{\hat{G}\left(t|{X}_i\right)}\right\}$$

Where $\hat{G}$ is the Kaplan-Meier estimate of the conditional survival function of the censoring time. Brier score value has a range of 0 and 1. Good predictions at time *t* denote small values. The integrated brier score (*IBS*) introduced by Graf is [32].

$$IBS=\frac{\int_0^{\mathit{\max}\left({t}_i\right)} BS(t) dt}{\mathit{\max}\left({t}_i\right)}$$

The smaller *IBS* value, the greater the prediction. Note that *IBS* has gradually become a standard evaluation measure for survival prediction methods and is commonly used in survival forests prediction [33].

*VIM* was computed just from the training set, whereas *IBS* and c-index were estimated in the test set. *IBS* and c-index are implemented in R package ***pec*** [34].

### Simulation result

#### Variable selection

**Fig. 1:**
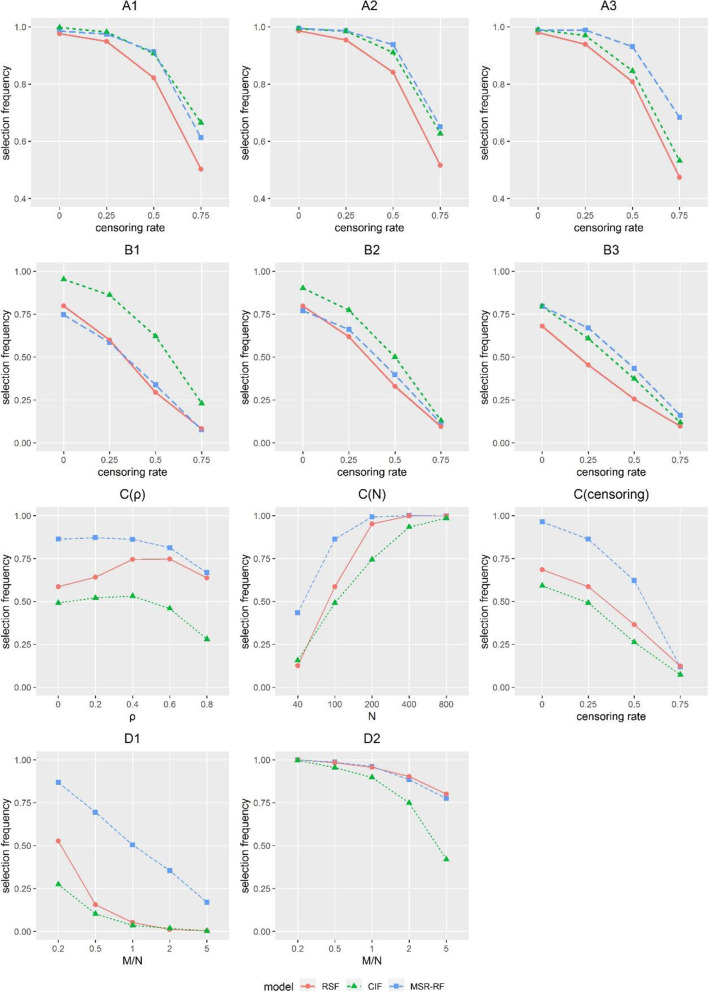
Correct variable selection frequency for datasets A-D with RSF, CIF and MSR-RF. In subplots A-B, sample size was fixed at 200. Dataset A was set in a linear form, whereas dataset B was set in an interaction term. The unordered-categorical covariate associated with the outcome was (A1, B1) covariate with 2 categories; (A2, B2) covariate with 4 categories; (A3, B3) covariate with 8 categories. Dataset C was set in a linear form with all ten variables generated form the multivariate normal distribution *MV*Ν(0, Σ). The subplot C(ρ) was fixed at *N* = 100 and 25% censoring; C(N) was fixed at ρ=0 and 25% censoring; C (censoring) was fixed at *N* = 100 and ρ=0. Dataset D was set in a linear form with all variables generated from the standard normal distribution for D1 and the binomial distribution with 0.5 probability for D2. The subplots D were fixed at *N* = 100 and 25% censoring with the various ratio *M/N*, which means the ratio of the number of covariates *M* to the sample size *N*

Figure 1 displays the ratio of identifying both the correct variables of models A-D.

Models A and B are presented as a function of different censoring rates at a fixed sample size of *N* = 200. In subplots A1, A2, A3, RSF have the lowest ratio of correct variable selection. CIF and MSR-RF vary slightly in A1 and A2, as CIF are a little bit better in A1 whereas conversely in A2. In A3, MSR-RF absolutely take over the lead. In subplots B1, B2, CIF perform best, while the others present equivalent performances. MSR-RF exhibit the best performance closely followed by CIF in B3 while RSF remain the poorest performance. More results are exhibited in Fig. S1 and S2. It can be seen that three methods all perform better when sample size increases, especially reach a nearly complete selection at a sample size of 800. RSF perform relatively badly in all situations. As models A and B share the same covariate framework, the results show that RSF may have a relatively weak ability in this kind of data, no matter with linear sets or interaction sets, which directs variable selection bias.

Dataset C was set in a linear form with all ten variables generated form the multivariate normal distribution *MV*Ν(0, Σ), and we use three subplots to describe it under different conditions. The subplot C(ρ) plots the results as a function of the correlation parameter ρ between the covariates at *N* = 100 and 25% censoring. It can be seen that the selection rate varies slightly when ρ < 0.6. The selection rate at ρ = 0.8 is lower than ρ = 0.6. C(N) plots the results of various sample sizes *N* at a fixed correlation parameter ρ=0 and 25% censoring, which means the covariates are independent. C(N) shows when the sample size *N* is smaller than 40, RSF and CIF both exhibit poor results with less than 20% selection rate. When *N* = 800, all three methods reach nearly complete variable selection. C (censoring) studies different censoring rates at *N* = 100 and ρ=0. It shows when the censoring rate is 75%, all three methods perform low selection rate less than 20%, but MSR-RF have absolutely higher selection rate under other censoring rates. In three subplots, it can be seen that the order of performance is MSR-RF, RSF, CIF in turn for most cases. More results are shown in Fig. S3, which verify the results above. The variable selection frequency doesn’t fluctuate too much for small correlations. CIF don’t perform well under this type of covariates.

The subplots D1, D2 plot the results as a function of number of covariates *M* at *N* = 100 and 25% censoring. In D1 with continuous covariates, MSR-RF offer obvious advantages across different dimensions of covariates. RSF are slightly better than CIF when *M/N* is no more than 1, which means sample size *N* is no less than the number of covariates *M*. When *M/N* > 1, both RSF and CIF perform badly, as the selection rate is nearly 0. In D2 with binary covariates, both MSR-RF and RSF perform much better than CIF when *M/N* increases. More results are shown in Fig. S4. In D1 MSR-RF show absolute advantages over the others. CIF are obviously weak, even sustain a selection rate of nearly 0 at a censoring rate of 75% no matter how *M/N* changes. MSR-RF closely follow RSF, and both methods have a selection rate of over 75% even when *M/N* = 5 in D2, while CIF perform conversely poor when *M/N* increases. As model D is aimed to study different numbers of covariates with continuous variables or categorical variables, the results show that CIF may not identify the correct covariates accurately in data with high dimensional covariates.

#### Prediction performance

**Fig. 2:**
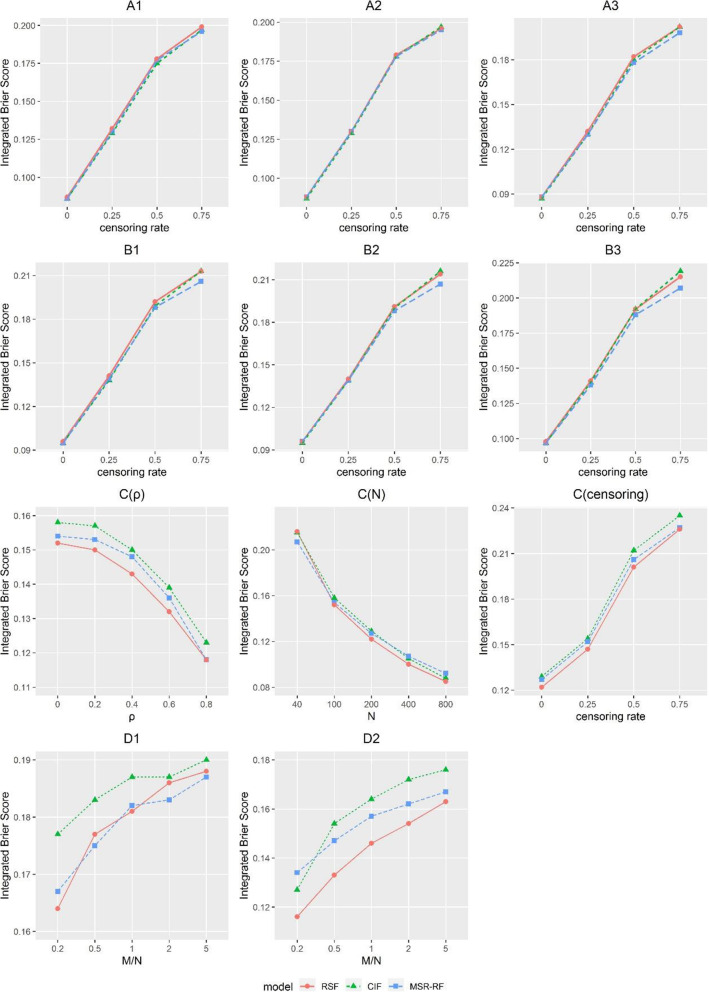
Integrated Brier score for datasets A-D with RSF, CIF and MSR-RF. In subplots A-B, sample size was fixed at 200. Dataset A was set in a linear form, whereas dataset B was set in an interaction term. The unordered-categorical covariate associated with the outcome was (A1, B1) covariate with 2 categories; (A2, B2) covariate with 4 categories; (A3, B3) covariate with 8 categories. Dataset C was set in a linear form with all ten variables generated from the multivariate normal distribution *MV*Ν(0, Σ). The subplot C(ρ) was fixed at *N* = 100 and 25% censoring; C(N) was fixed at ρ=0 and 25% censoring; C (censoring) was fixed at *N* = 100 and ρ=0. Dataset D was set in a linear form with all variables generated from the standard normal distribution for D1 and the binomial distribution with 0.5 probability for D2. The subplots D were fixed at *N* = 100 and 25% censoring with the various ratio *M/N*, which means the ratio of the number of covariates *M* to the sample size *N*

Figure 2 displays the mean value of *IBS* of models A-D, with the same parameter settings as Fig. 1.

Models A and B were investigated as a function of different censoring rates at a fixed sample size of *N* = 200. In subplots A1-A3, all three curves almost coincide. In subplots B1-B3, MSR-RF perform better under 75% censoring, whereas three methods remain overlapped when censoring rate less than 50%. More results in Fig. S5 and S6 prove the findings above. The results of c-index in Fig. S9 and S10 also indicate that there are only slight differences within 0.02 between the curves at the same settings, so it’s hard to conclude which perform best.

The subplot C(ρ) was fixed at *N* = 100 and 25% censoring, and *IBS* decreases as ρ increases. RSF perform slightly better, followed by MSR-RF and lastly CIF. C(N) was fixed at ρ=0 and 25% censoring. It shows MSR-RF performs best when *N* = 40, and RSF take over the lead when *N* > 40. C (censoring) was fixed at *N* = 100 and ρ=0, in which RSF maintain the best prediction. Overall three curves only have small *IBS* gap and Fig. S7 proves it. The results of c-index in Fig. S11 indicate that MSR-RF are superior. RSF perform poor as CIF when *N* = 40, whereas RSF perform just a little bit lower than MSR-RF when *N* > 40. Overall, CIF remain the poorest performance.

The subplots D1, D2 plot the results as a function of number of covariates *M* at *N* = 100 and 25% censoring. *IBS* increases as the ratio *M/N* increases. In D1 with continuous covariates, both MSR-RF and RSF have lower *IBS* than CIF. In D2 with binary covariates, RSF offer obvious advantages across different *M*. Same findings can be observed in Fig. S8 with more results. The results of c-index in Fig. S12 indicate that MSR-RF are superior in D, followed by RSF and lastly CIF. In D2 with binary covariates, RSF perform just a little bit lower than MSR-RF. Overall, CIF still remain the poorest performance.

## Application study

To demonstrate the efficiency and the predictive performance of the three survival forest models, we analyzed two real datasets with MSR-RF, RSF and CIF. For forest construction, 200 survival trees were grown for each survival forest. In each simulation, we randomly selected 80% subjects as training set and the other 20% as test set, and this was repeated 100 times. For each repetition, *IBS* of test set were recorded and shown as boxplots. For easy explanation, Cox model was also conducted with the same analysis as a benchmark model.

The lung dataset recorded survival in patients with advanced lung cancer from the North Central Cancer Treatment Group (NCCTG) [35]. Subjects with missing values were excluded, so 167 subjects with 8 covariates were retained for analysis in our study. The median survival time is 268(range: 5 ~ 1022) days. A total of 120 patients died, with a low censoring rate of 28.1%. Summary characteristics can be found in Table 2. The dataset has 5 continuous covariates and 3 categorical covariates, including one with 2 categories, one with 4 categories and one with 17 categories. The dataset is freely available in the R package ***survival*** [36].

**Table 2:** Characteristics of the covariates in Dataset lung [Mean ± SD or *n* (%)]

Characteristics	Total(*n* = 167)
inst (institution code)	
1	28 (16.8)
2	4 (2.4)
3	12 (7.2)
4	4 (2.4)
5	7 (4.2)
6	12 (7.2)
7	7 (4.2)
10	4 (2.4)
11	13 (7.8)
12	16 (9.6)
13	13 (7.8)
15	6 (3.6)
16	10 (6.0)
21	8 (4.8)
22	13 (7.8)
26	4 (2.4)
32	6 (3.6)
sex	
male	103 (61.7)
female	64 (38.3)
ph.ecog (ECOG performance score rated by physician)	
asymptomatic	47 (28.1)
symptomatic but completely ambulatory	81 (48.5)
in bed < 50% of the day	38 (22.8)
in bed > 50% of the day but not bedbound	1 (0.6)
age	62.57 ± 9.21
ph.karno (Karnofsky performance score rated by physician)	82.04 ± 12.78
pat.karno (Karnofsky performance score rated by patient)	79.58 ± 15.10
meal.cal (Calories consumed at meals)	929.13 ± 413.49
wt.loss (Weight loss in last six months)	9.72 ± 13.38

**Fig. 3:**
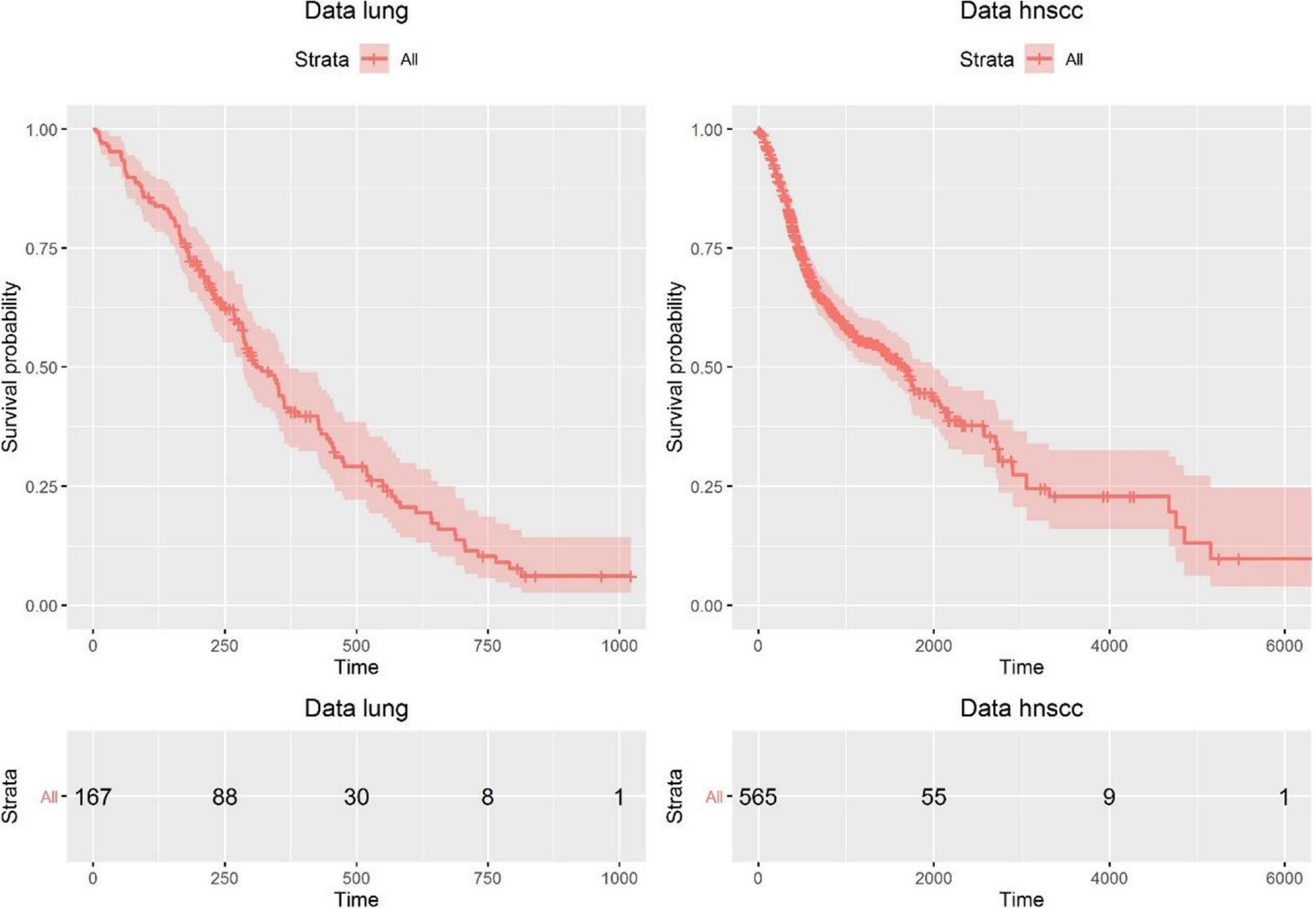
Survival curve generated from lung dataset and hnscc dataset

The *hnscc* dataset is a high dimensional breast cancer gene expression data with 565 subjects and 99 continuous covariates. The median survival time is 1671 (range: 2–6417) days. A total of 253 patients died, with a censoring rate of 55.2%. The dataset is freely available in the R package *SurvHiDim* [37]. Survival curves generated from lung dataset and hnscc dataset are shown in Fig. 3.

**Fig. 4:**
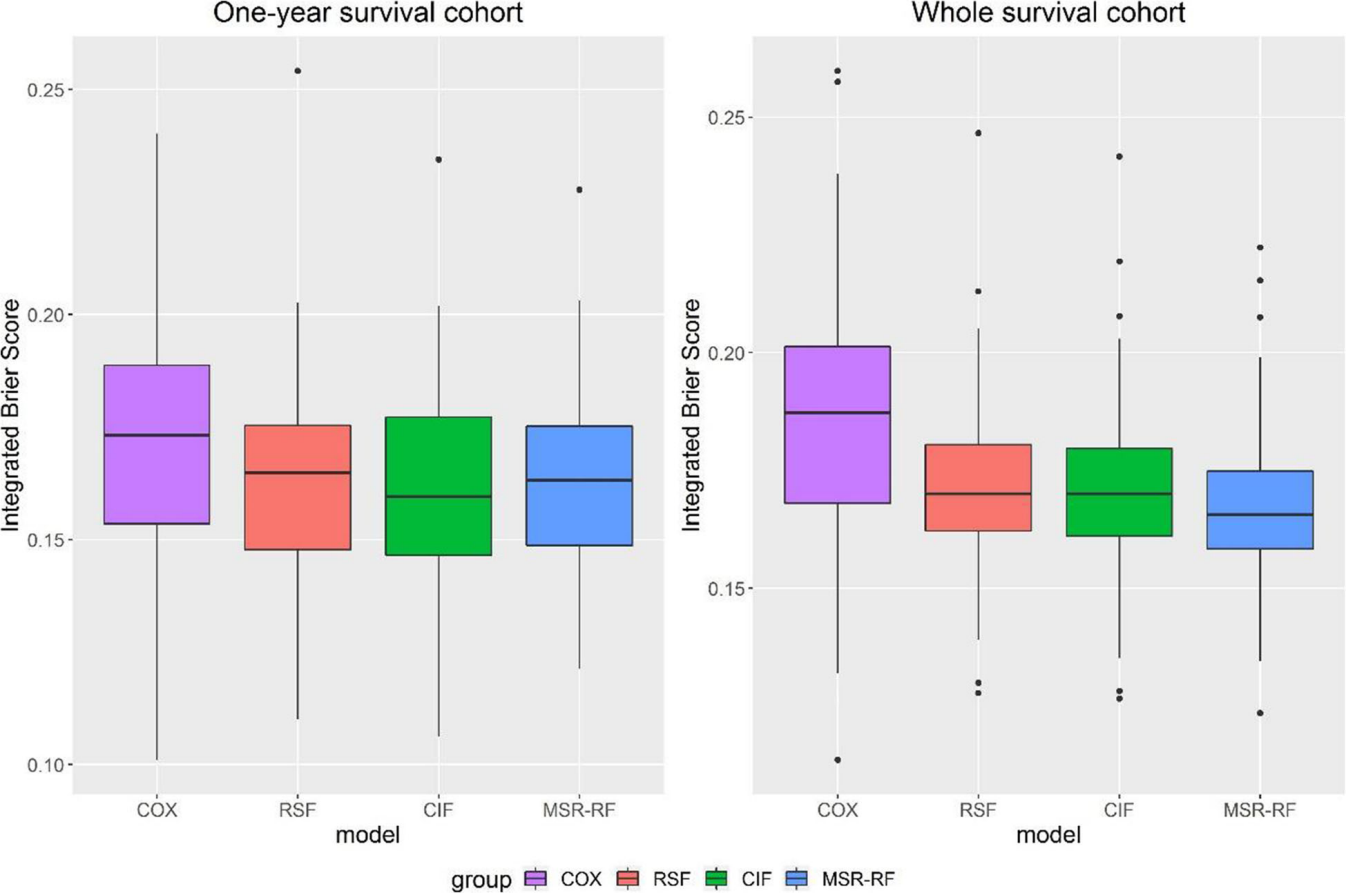
Integrated Brier scores of lung dataset

**Fig. 5:**
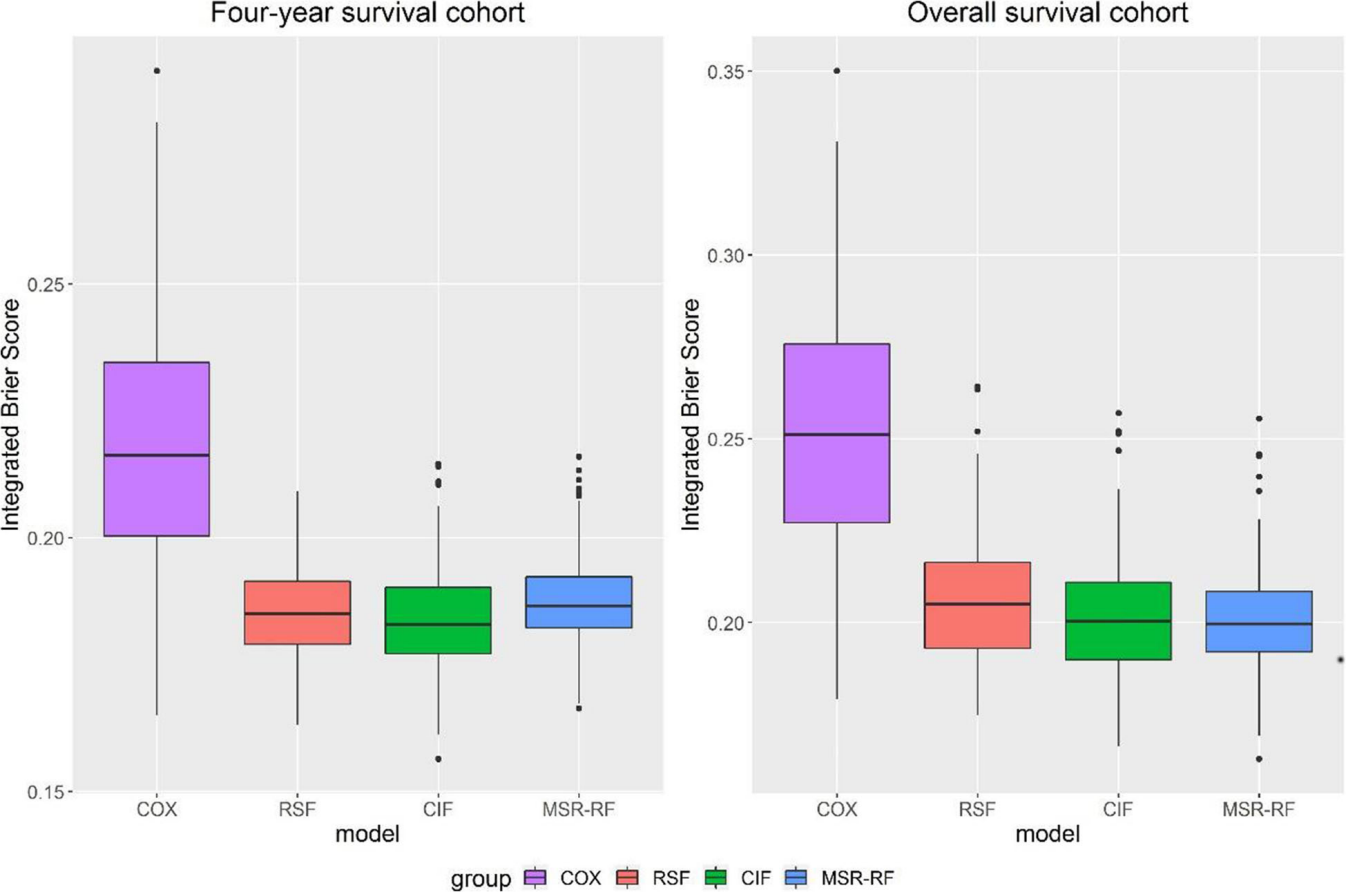
Integrated Brier scores of hnscc dataset

Besides the overall survival, researchers may also have interest in survival of specific time. In this way, despite the overall survival cohort of each dataset, we also present the 1-year survival prediction of lung dataset and the 4-year survival prediction of hnscc dataset. In Figs. 4 and 5, we find that all three forests perform better than the default benchmark Cox model. For lung dataset, all three forests seem to be comparable in predicting 1-year survival while CIF have the lowest median value. MSR-RF show the relatively low *IBS* range in overall survival prediction. What’s more, MSR-RF seem to be the most stable method here because of the smallest range and interquartile range. For hnscc dataset, MSR-RF show the smallest range and interquartile range in both four-year survival prediction and overall survival prediction whereas CIF show the largest conversely.

**Fig. 6:**
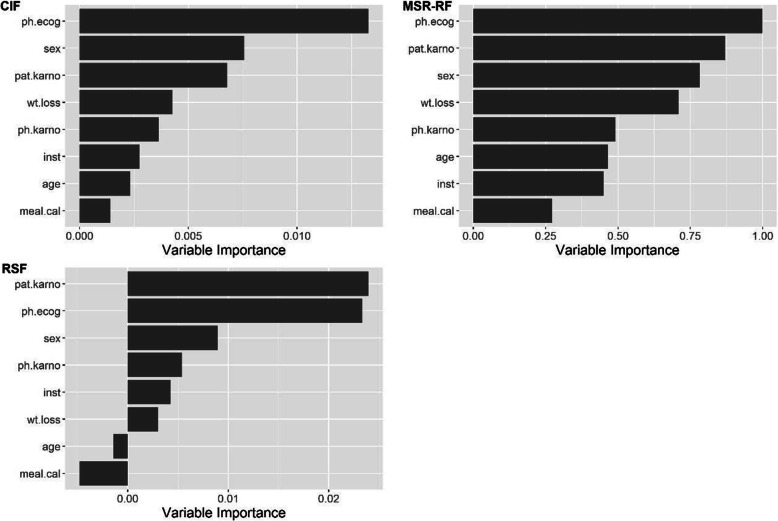
Variable importance on Dataset lung

Figure 6 presents the variable importance result of lung dataset. CIF and MSR-RF have similar results in identifying the factors affecting the survival outcome, as ph.ecog, wt.loss, ph.karno, meal.cal rank 1st, 4th, 5th, 8th respectively in both forests. Sex, pat.karno rank 2nd-3rd and inst, age rank 6th–7th in both forests with slight difference in order. However, despite the difference in RSF, ph.ecog, sex, pat.karno are the top three predictors among all methods, and meal.cal tends to have the lowest association with the outcome.

For the variable selection performance of hnscc dataset, unlike the custom establishment of correct variables in the simulation part, we have to use other variable selection method to learn the variables as a reference. Here we used backward stepwise selection based on AIC criterion as a reference and 38 covariates were selected. Ranks of *VIM* of the 38 variables were calculated among all the 99 variables. Median ranks of *VIM* and frequency of the selected variables’ *VIM* ranking top 38 of all repetitions were listed in Table 3. It can be seen that three methods differ in the ranks. CIF have the largest range and interquartile range, which indicate a relatively dispersion in the result. RSF and MSR-RF have a relatively close and robust performance compared to CIF.

**Table 3:** The ranks of variable importance on hnscc dataset

Characteristics	Median ranks of *VIM*			Frequency of *VIM* ranking top 38		
	RSF	CIF	MSR-RF	RSF	CIF	MSR-RF
AFF3	9	5	8	0.81	0.99	0.85
PCDP1	49	36.5	52	0.43	0.54	0.35
ADH1B	12	23.5	39.5	0.87	0.8	0.48
RIMKLA	63	80.5	64.5	0.21	0.07	0.12
PCDHA12	58	64	56.5	0.24	0.21	0.14
CDHR5	48	11.5	32	0.43	0.94	0.56
PGC	4	19.5	37.5	0.94	0.87	0.51
NME5	48	43	45	0.31	0.43	0.43
XIST	36.5	27.5	50	0.51	0.66	0.33
B3GAT1	15	17.5	19	0.72	0.91	0.75
C1orf88	14	6	5	0.8	0.97	0.98
SELENBP1	17	25	15.5	0.73	0.77	0.75
ASRGL1	56.5	41.5	39	0.36	0.47	0.5
SGK2	52	15.5	45.5	0.36	0.86	0.44
PPP1R9A	49	81.5	66	0.29	0.05	0.21
KLF15	48	54.5	50	0.34	0.3	0.35
PNMA2	67	74	56.5	0.16	0.08	0.26
DNALI1	45	58	36	0.38	0.29	0.53
TMEM178	62.5	74	58.5	0.28	0.08	0.25
GAL3ST1	31.5	9	17	0.57	0.93	0.71
PLCXD3	42.5	64	55.5	0.47	0.13	0.19
RSPH1	44.5	24.5	48	0.44	0.7	0.4
METTL7B	31	17	25	0.58	0.88	0.62
DCDC2	63.5	64	61.5	0.17	0.19	0.16
DDAH1	64.5	56	44	0.24	0.26	0.46
RGL3	58.5	72	59.5	0.23	0.1	0.18
C16orf89	54.5	77	62	0.31	0.05	0.26
TCF21	61.5	66	51.5	0.24	0.16	0.33
C8orf46	62.5	70	66	0.21	0.11	0.32
CTSE	23	14	10	0.67	0.9	0.81
GJB1	60	77	64	0.18	0.06	0.22
BEX1	31	28	26.5	0.6	0.67	0.56
ABCC6P2	57	46.5	46	0.31	0.38	0.44
MYEF2	45.5	37	44	0.46	0.52	0.43
SLC4A4	63.5	62	61	0.18	0.19	0.3
CA8	64.5	68	61.5	0.23	0.13	0.19
CACNA1D	44	43	41	0.41	0.43	0.45
AQP4	62	77.5	58	0.2	0.06	0.22
median	48.5	44.75	47	0.36	0.40	0.42
range	4 ~ 67	5 ~ 81.5	5 ~ 66	0.16 ~ 0.94	0.05 ~ 0.99	0.12 ~ 0.98
IQR	32.75 ~ 61.12	23.75 ~ 67.5	36.38 ~ 58.38	0.24 ~ 0.56	0.13 ~ 0.79	0.25 ~ 0.53

## Discussion and conclusion

In this paper we used simulation study and real data study to compare prediction performances and variable selection performances between three survival forests mentioned above, including RSF, CIF and MSR-RF. The prediction performance was evaluated through the prediction error *IBS* based on brier score with c-index as supplement. The smaller *IBS* value, the greater the prediction. The variable selection performance was evaluated by calculating the frequency of the correct variables ranking in the top by *VIM.*

The variable selection performance in simulation study shows that CIF and MSR-RF both outperform RSF when there are multiple categorical variables in the datasets, where CIF show advantages in dealing with interaction term. When there are only continuous variables in the datasets, MSR-RF perform better. When there are only binary variables in the data, RSF and MSR-RF are superior than CIF. The results also show that three forests methods are not sensitive to the correlation between covariates due to the fact that correlation degree of the variables has little effect on the selection frequency. When the variable dimension increases, MSR-RF and RSF seem to be more robust than CIF.

However, the *IBS* results and c-index results in predictive performance show that three methods are comparable majorly, only small variations can be observed under some situations. When there are only continuous variables or binary variables in the datasets, MSR-RF and RSF seem to perform better than CIF. The results in application study are similar to those from simulation study. All three forest methods outperform the benchmark Cox model based on *IBS* result, but there are only tiny differences among the three methods. MSR-RF seem to be more stable based on smaller range and interquartile range.

What’s more, it can be seen that higher correct variable selection frequency does not match better *IBS* or c-index value exactly in simulation study, which indicates prediction performance and variable selection performance are worth taking into consideration respectively, and that’s the objective in this paper.

There are several limitations in our study. First, high dimensional datasets have been considered only in model D in our research, which studied only continuous or binary variables. We have to admit that we lack deep investigations in high or ultra-high dimensional datasets in this paper because we think it’s a wide field deserving deep investigations and we will make independent research in the future. Next, our paper studied both variable selection performance and prediction performance respectively. The prediction performance of simulation and application is easy to exhibit, which has been done in previous comparative studies. However, the variable selection performance of application is hard to evaluate because the correct variables associated to the outcome are unknown, whereas they can be set in simulation. We can only learn those variables from other variable selection methods for real datasets such as stepwise selection, LASSO et al. to serve as reference. Nevertheless, no method could be regarded as a “gold standard” reference. In this paper we just conducted the backward stepwise selection and exhibited the distribution of the ranks of *VIM*.

The main finding of this study is that RSF, CIF, MSR-RF all show advantages based on different type of covariates. Hence it’s important for researchers to choose an appropriate forest model according to the research aim (variable selection or better prediction) and the nature of covariates. As it is shown in our study, MSR-RF exhibit a relatively good and stable performance in most situations. Years ago the proposers conducted studies on computational time and proved the realization faster, which is also observed in our study. In this way, MSR-RF are worth generalization and we hope the method could raise more attention in biomedical field.

## Supplementary Information

**Additional file 1.**

## Data Availability

The authors confirm that all data underlying the findings are fully available without restriction. Dataset lung analysed during the current study is publically available from https://ascopubs.org/doi/10.1200/JCO.1994.12.3.601. Dataset hnscc analysed during the current study is publically available from https://CRAN.R-project.org/package=SurvHiDim.

## Abbreviations

RSF: random survival forests
CIF: conditional inference forests
MSR-RF: random forests with maximally selected rank statistics
IBS: integrated Brier score
VIM: variable importance measure
Cox model: Cox-proportional hazards regression model
RF: random forests
OOB data: out-of-bag data
CHF: cumulate hazard function
SF: survival function
CIT: conditional inference tree
MSR: maximally selected rank statistics
NCCTG: North Central Cancer Treatment Group
