# Supplementary material for: A comparative study of forest methods for time-to-event data: variable selection and predictive performance
Source: BMC Med Res Methodol. 2021 Sep 25;21:193. doi: 10.1186/s12874-021-01386-8 (PMC8465777; doi:10.1186/s12874-021-01386-8)
Supplement: Supplementary file 1 — Additional file 1. [file 12874_2021_1386_MOESM1_ESM.docx]

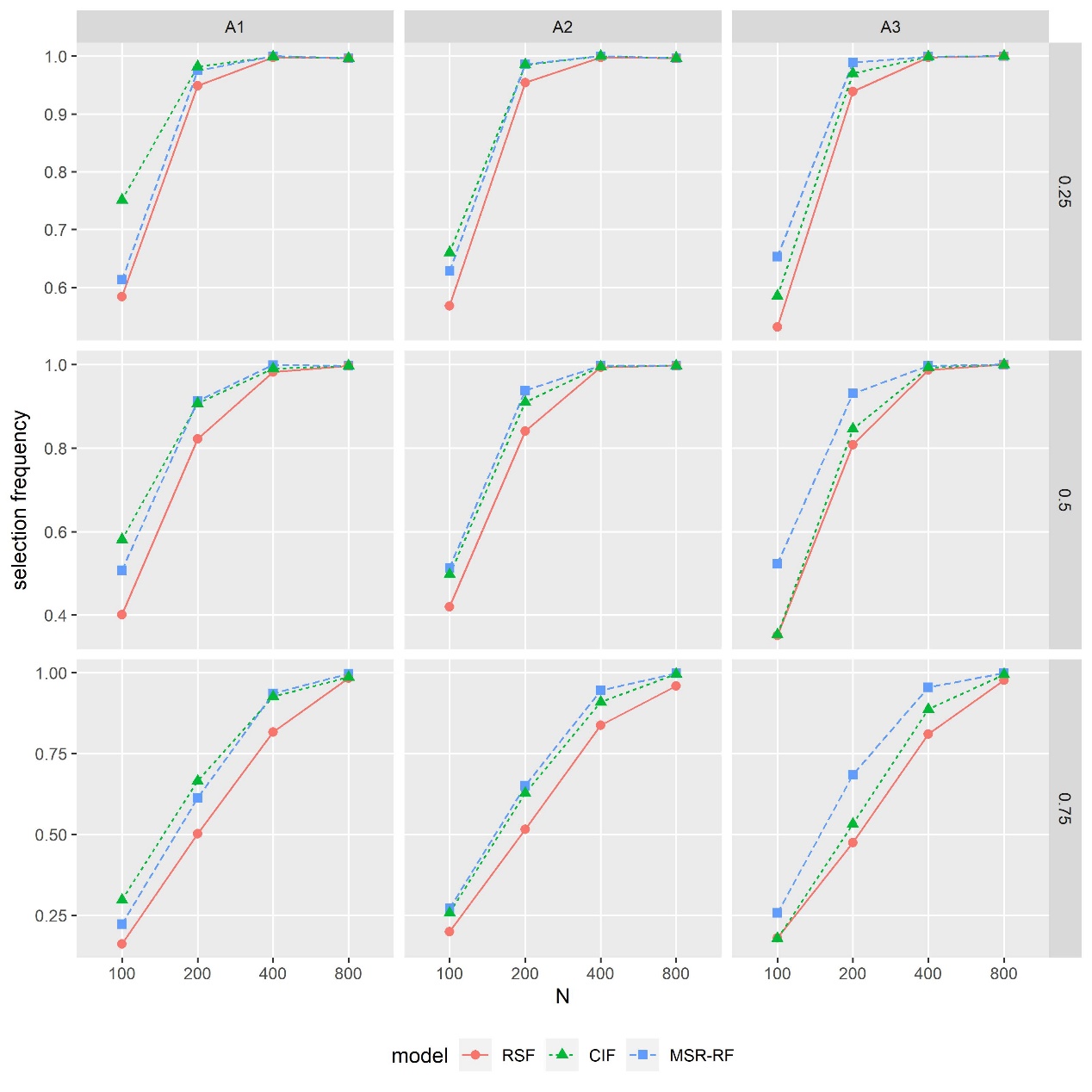


Fig S1 Correct variable selection frequency for datasets A with RSF, CIF and MSR-RF.

Dataset A was set in a linear form, with a continuous variable and a categorical variable associated with the outcome. The categorical covariate associated with the outcome was (A1) covariate with 2 categories; (A2) covariate with 4 categories; (A3) covariate with 8 categories. The sample size *N* was set to 100,200,400,800; the censoring rate was set to 25%, 50%, 75%.


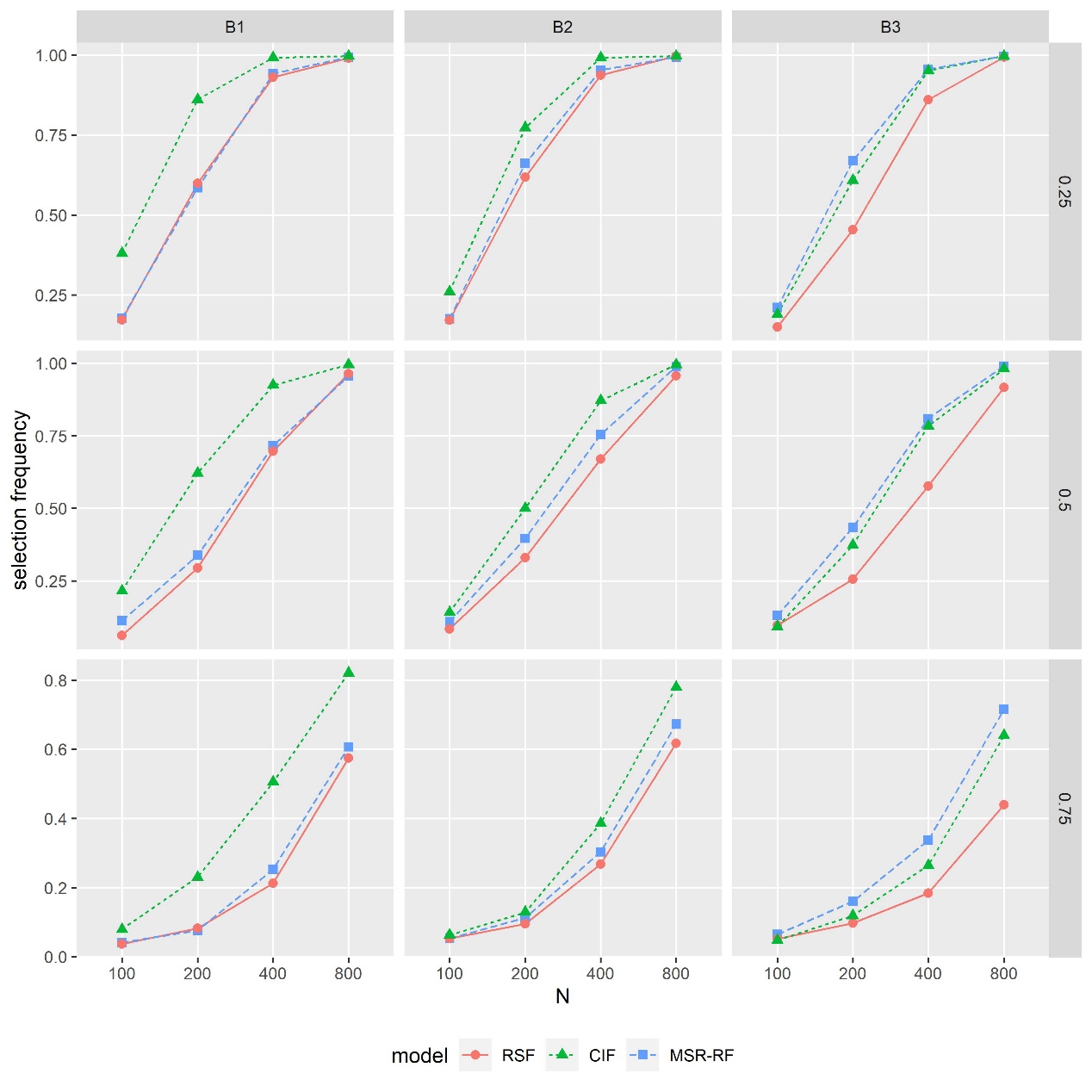


Fig S2 Correct variable selection frequency for datasets B with RSF, CIF and MSR-RF.

Dataset B was set in an interaction form, with a continuous variable and a categorical variable associated with the outcome with coefficient 1. The categorical covariate associated with the outcome was (B1) covariate with 2 categories; (B2) covariate with 4 categories; (B3) covariate with 8 categories. The sample size *N* was set to 100,200,400,800; the censoring rate was set to 25%, 50%, 75%.


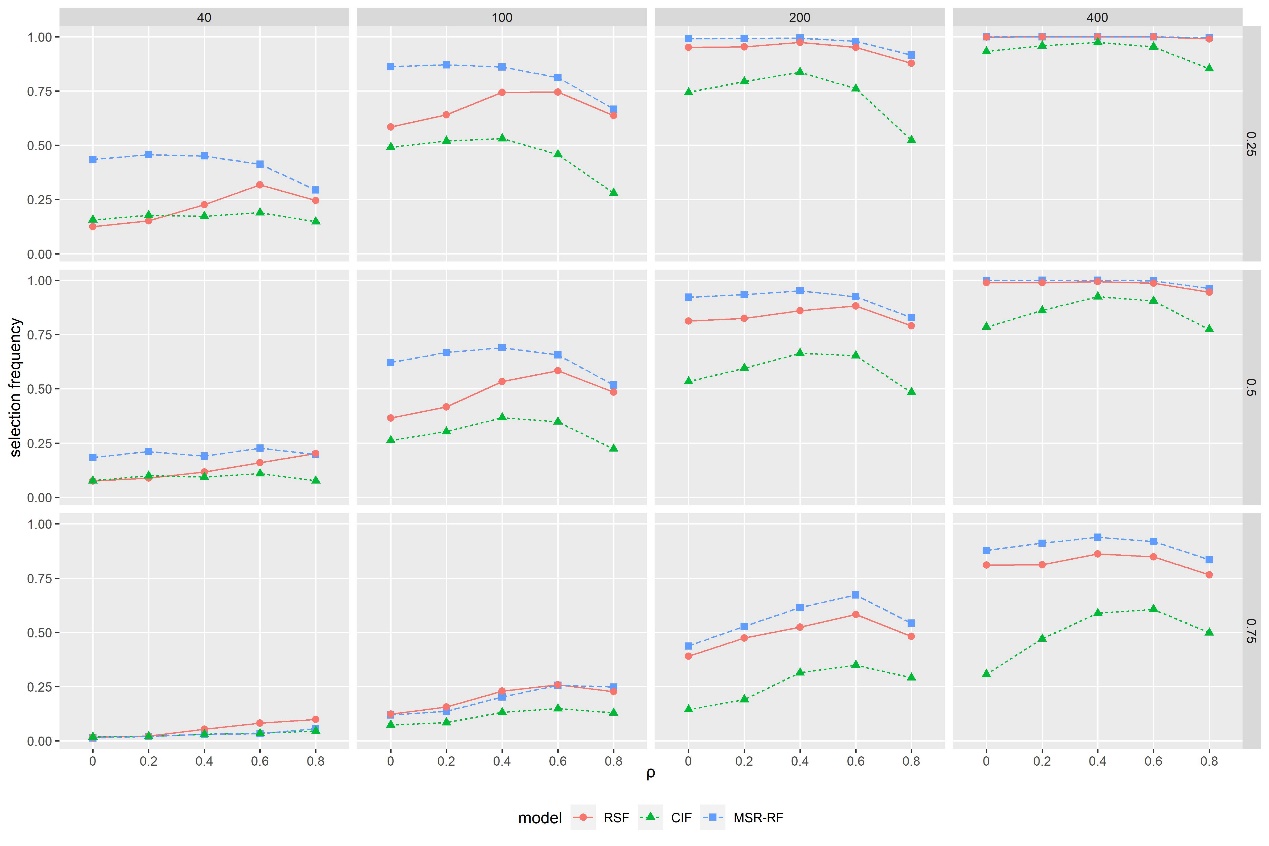


Fig S3 Correct variable selection frequency for datasets C with RSF, CIF and MSR-RF.

Dataset C was set in a linear form with all ten variables generated from the multiple normal distribution. Selection frequency means the rates of the two correct variables $x_{1}$ and $x_{2}$ ranking top, whereas $x_{1}$ with coefficient 1 and $x_{2}$ with coefficient 1.5. The sample size *N* was set to 40, 100,200,400; the censoring rate was set to 25%, 50%, 75%. The correlation parameter $\rho$ was set to 0, 0.2, 0.4, 0.6, 0.8.


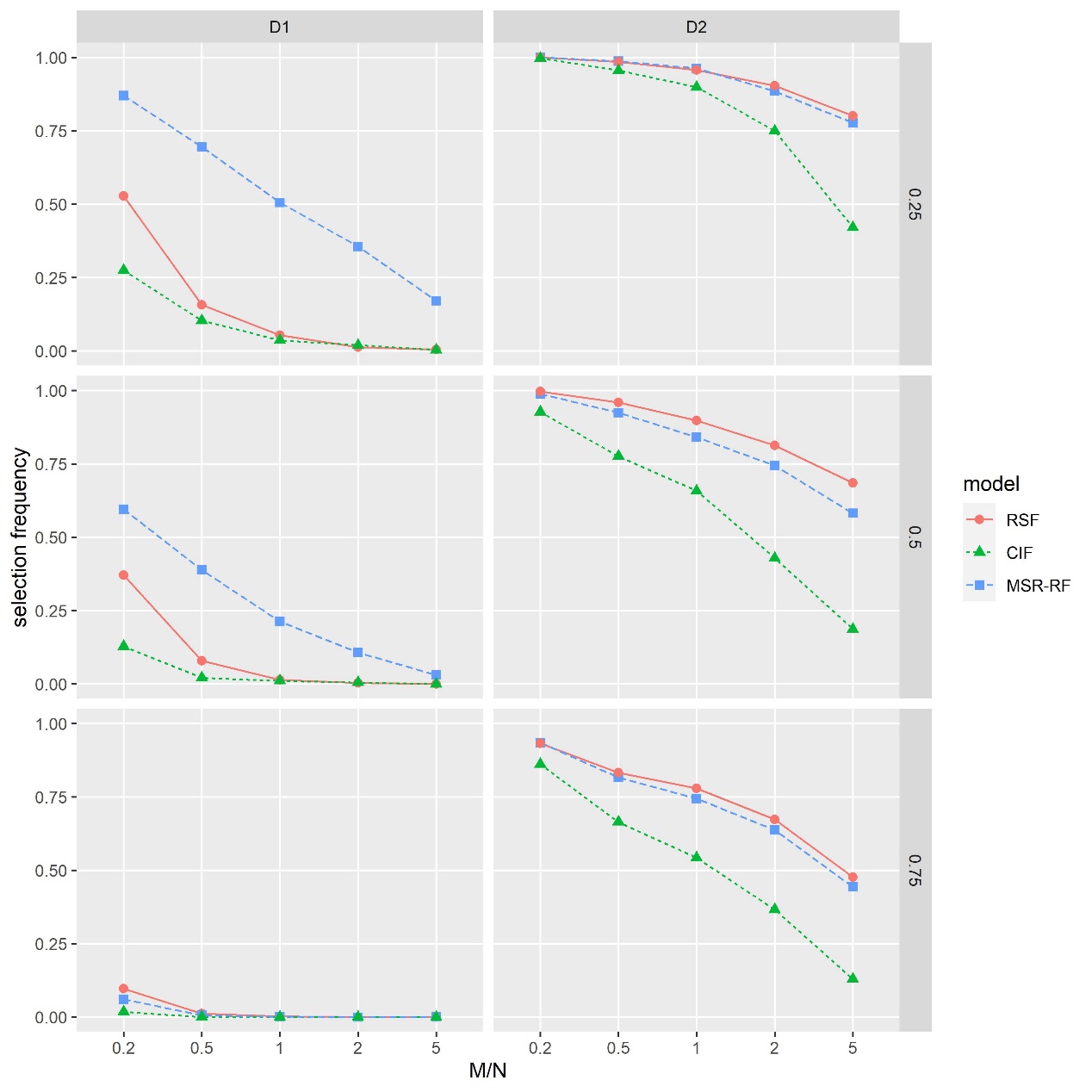


Fig S4 Correct variable selection frequency for datasets D with RSF, CIF and MSR-RF.

Dataset D was set in a linear form with all variables generated from the standard normal distribution for D1 and the binomial distribution with 0.5 probability for D2. Selection frequency means the rates of the two correct variables $x_{1}$ and $x_{2}$ranking top, whereas $x_{1}$ with coefficient 2 and $x_{2}$ with coefficient 3. The sample size *N* was set to 100; the censoring rate was set to 25%, 50%, 75%. The ratio *M/N*, which means the ratio of the number of covariates *M* to the sample size *N*, was set to 0.2, 0.5, 1, 2, 5.


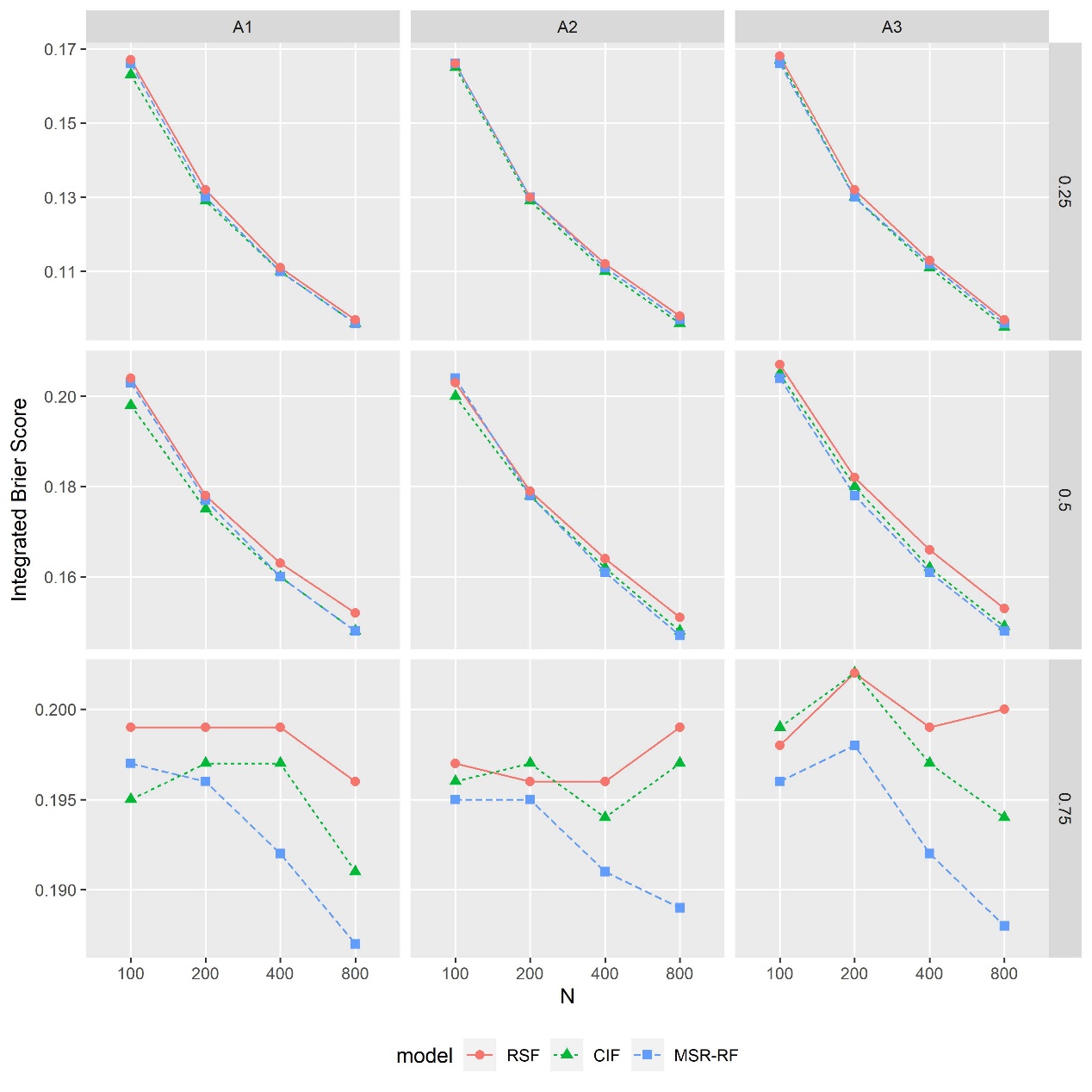


Fig S5 Integrated Brier scores for datasets A with RSF, CIF and MSR-RF.

Dataset A was set in a linear form, with a continuous variable and a categorical variable associated with the outcome. The categorical covariate associated with the outcome was (A1) covariate with 2 categories; (A2) covariate with 4 categories; (A3) covariate with 8 categories. The sample size *N* was set to 100,200,400,800; the censoring rate was set to 25%, 50%, 75%.


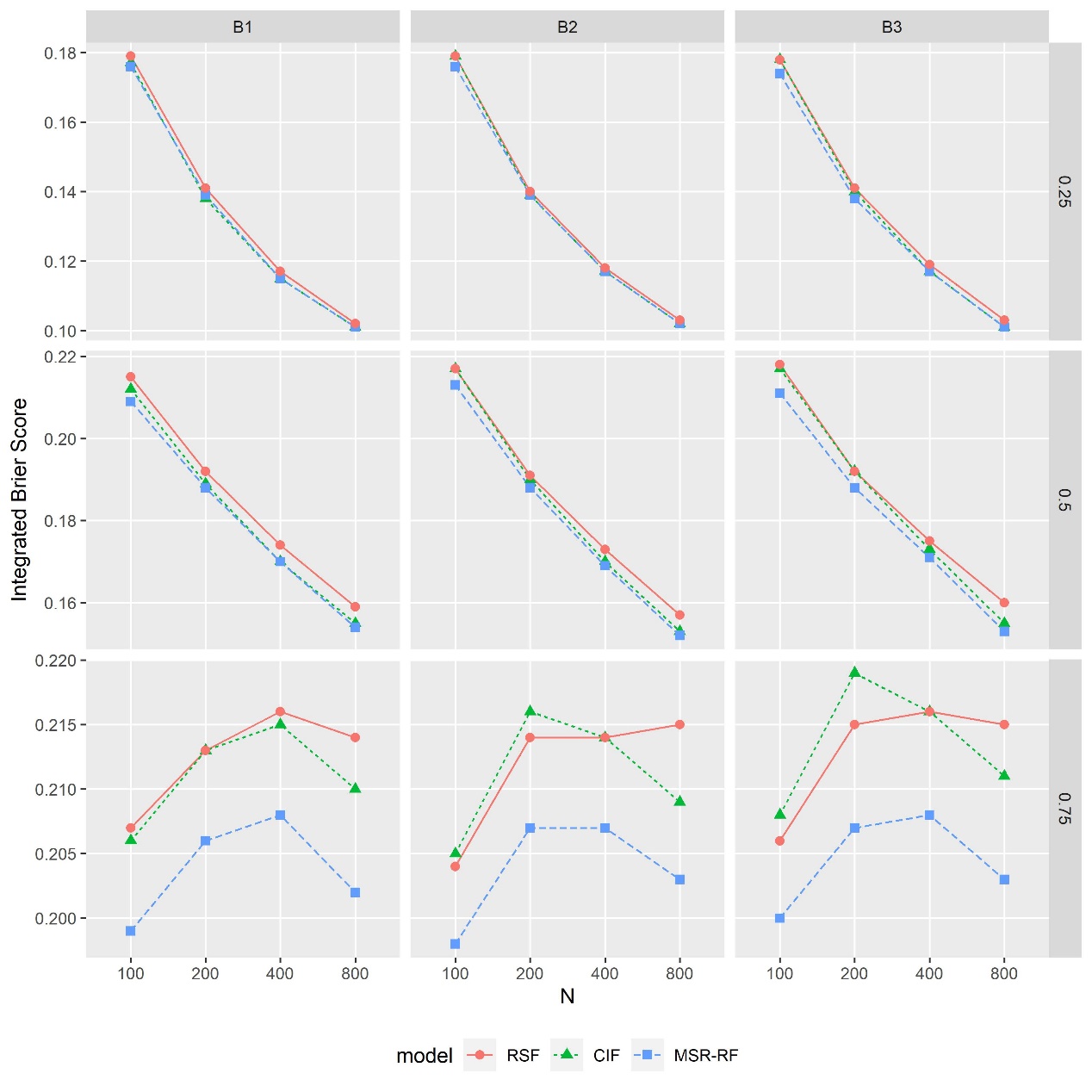


Fig S6 Integrated Brier scores for datasets B with RSF, CIF and MSR-RF.

Dataset B was set in an interaction form, with a continuous variable and a categorical variable associated with the outcome with coefficient 1. The categorical covariate associated with the outcome was (B1) covariate with 2 categories; (B2) covariate with 4 categories; (B3) covariate with 8 categories. The sample size *N* was set to 100,200,400,800; the censoring rate was set to 25%, 50%, 75%.


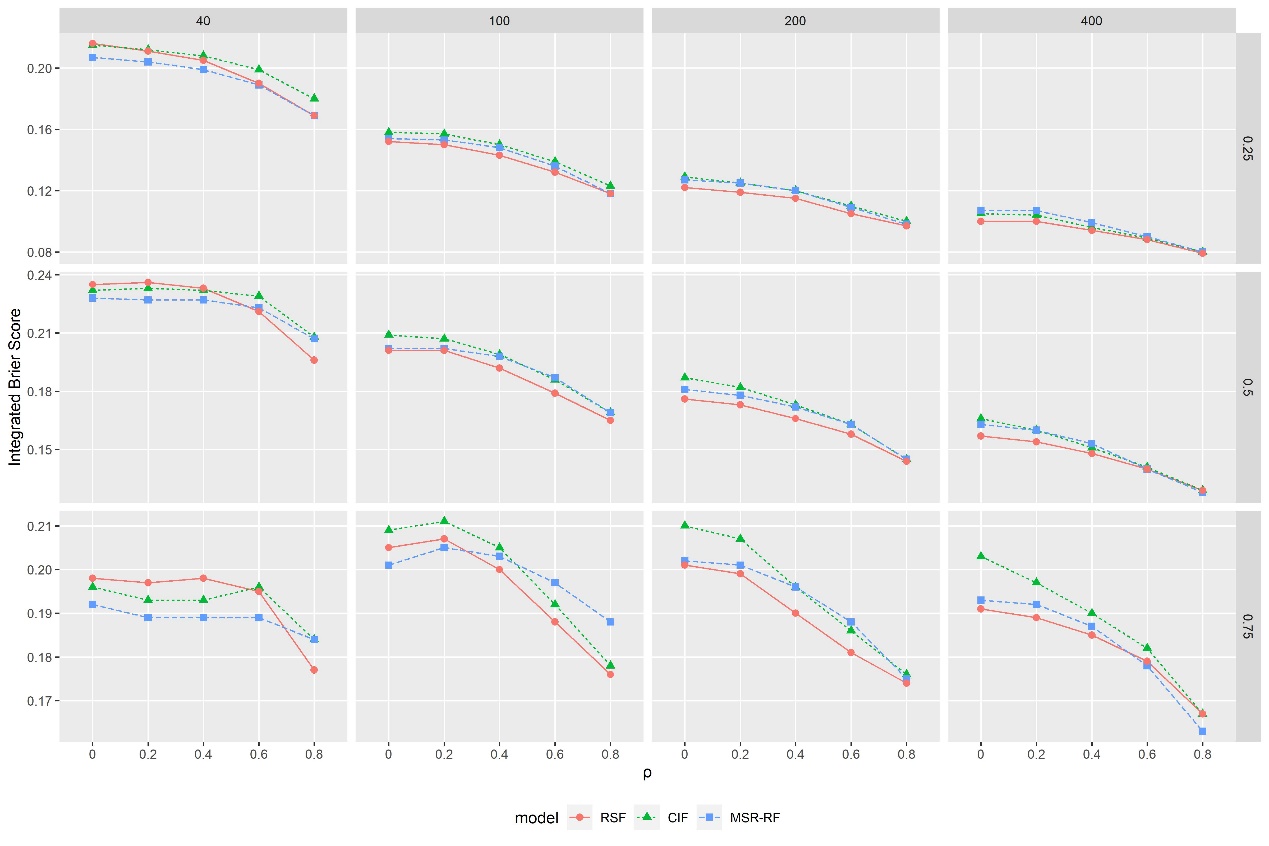


Fig S7 Integrated Brier scores for datasets C with RSF, CIF and MSR-RF.

Dataset C was set in a linear form with all ten variables generated from the multiple normal distribution. Selection frequency means the rates of the two correct variables $x_{1}$ and $x_{2}$ ranking top, whereas $x_{1}$ with coefficient 1 and $x_{2}$ with coefficient 1.5. The sample size *N* was set to 40, 100,200,400; the censoring rate was set to 25%, 50%, 75%. The correlation parameter $\rho$ was set to 0, 0.2, 0.4, 0.6, 0.8.


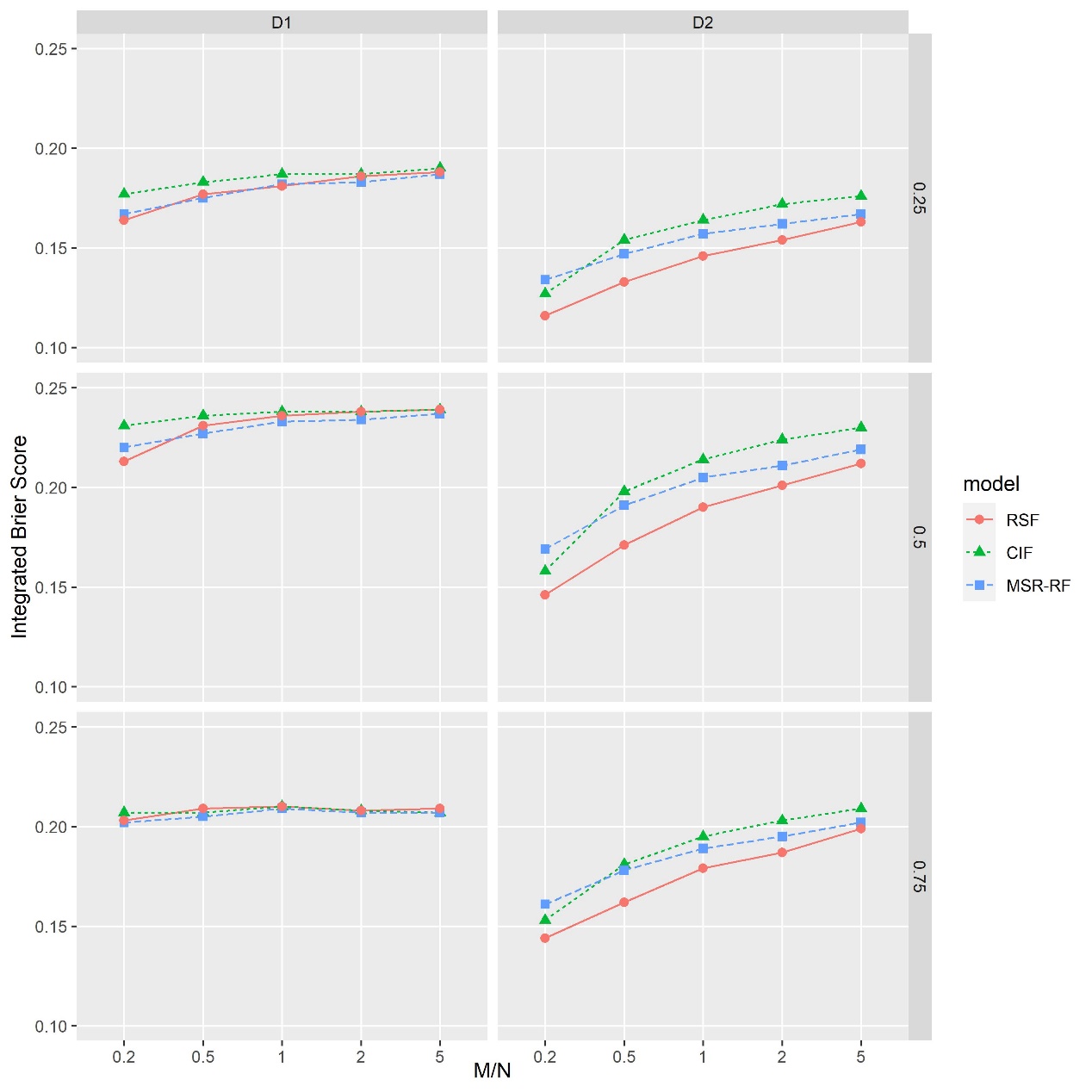


Fig S8 Integrated Brier scores for D datasets with RSF, CIF and MSR-RF.

Dataset D was set in a linear form with all variables generated from the standard normal distribution for D1 and the binomial distribution with 0.5 probability for D2. Selection frequency means the rates of the two correct variables $x_{1}$ and $x_{2}$ranking top, whereas $x_{1}$ with coefficient 2 and $x_{2}$ with coefficient 3. The sample size *N* was set to 100; the censoring rate was set to 25%, 50%, 75%. The ratio *M/N*, which means the ratio of the number of covariates *M* to the sample size *N*, was set to 0.2, 0.5, 1, 2, 5.


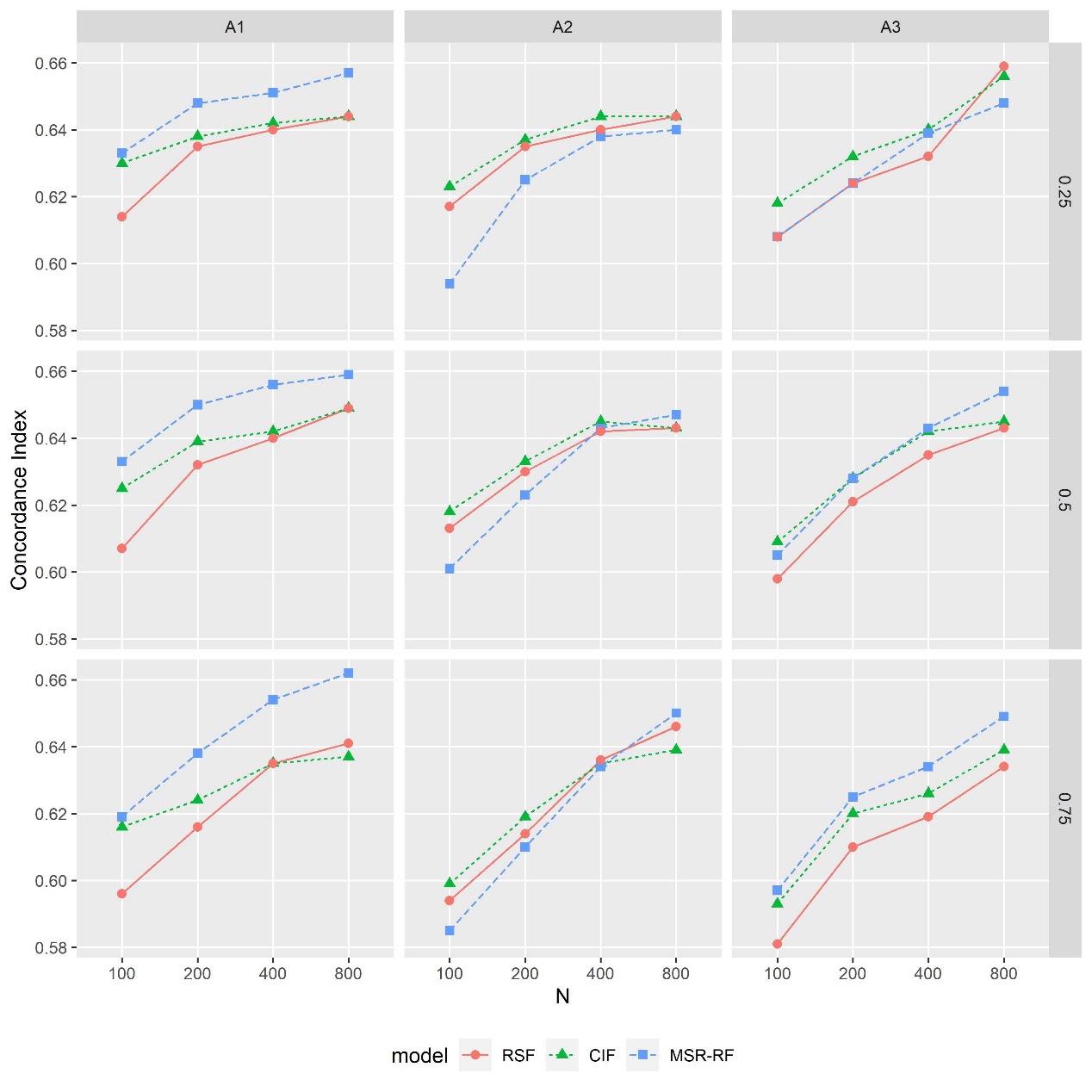


Fig S9 Concordance index for datasets A with RSF, CIF and MSR-RF.

Dataset A was set in a linear form, with a continuous variable and a categorical variable associated with the outcome. The categorical covariate associated with the outcome was (A1) covariate with 2 categories; (A2) covariate with 4 categories; (A3) covariate with 8 categories. The sample size *N* was set to 100,200,400,800; the censoring rate was set to 25%, 50%, 75%.


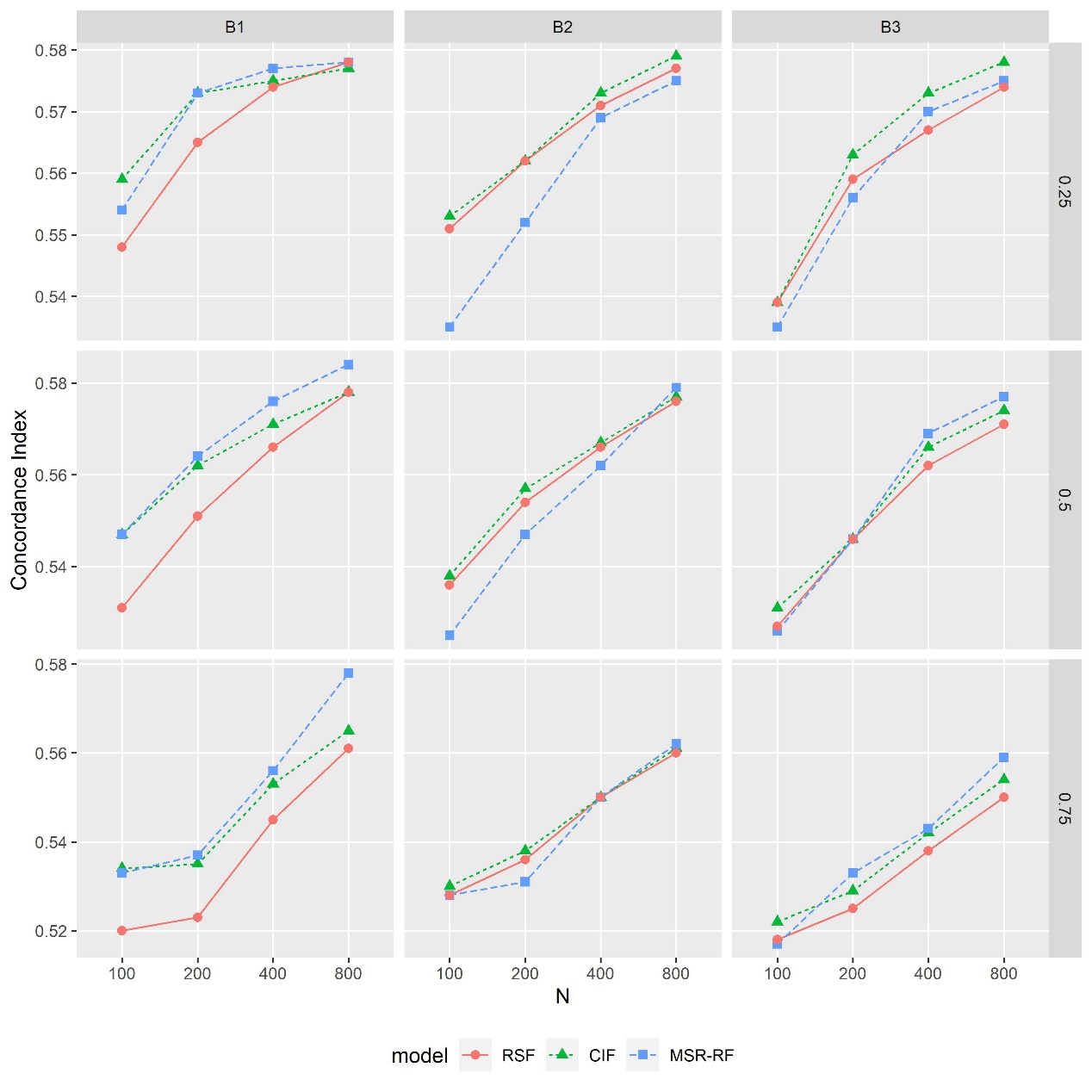


Fig S10 Concordance index for datasets B with RSF, CIF and MSR-RF.

Dataset B was set in an interaction form, with a continuous variable and a categorical variable associated with the outcome with coefficient 1. The categorical covariate associated with the outcome was (B1) covariate with 2 categories; (B2) covariate with 4 categories; (B3) covariate with 8 categories. The sample size *N* was set to 100,200,400,800; the censoring rate was set to 25%, 50%, 75%.


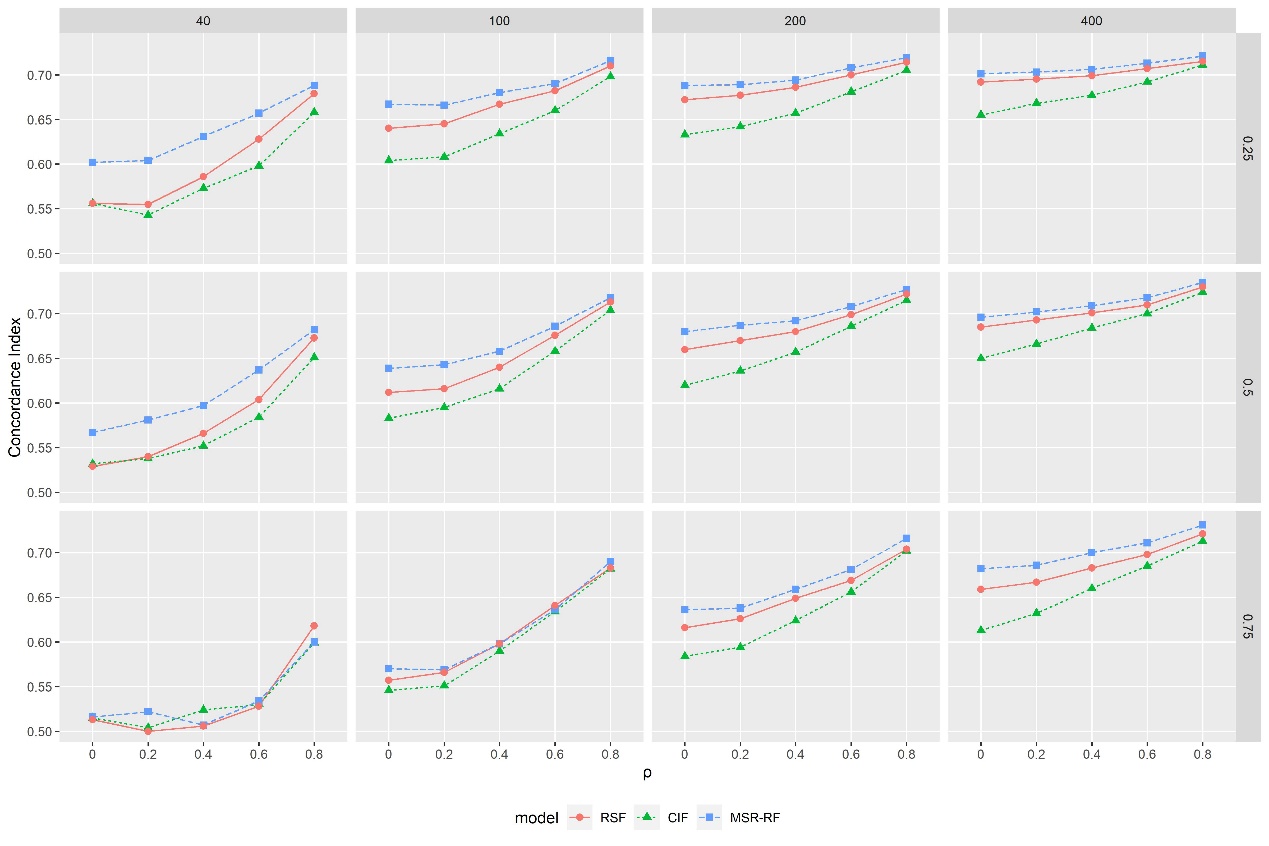


Fig S11 Concordance index for datasets C with RSF, CIF and MSR-RF.

Dataset C was set in a linear form with all ten variables generated from the multiple normal distribution. Selection frequency means the rates of the two correct variables $x_{1}$ and $x_{2}$ ranking top, whereas $x_{1}$ with coefficient 1 and $x_{2}$ with coefficient 1.5. The sample size *N* was set to 40, 100,200,400; the censoring rate was set to 25%, 50%, 75%. The correlation parameter $\rho$ was set to 0, 0.2, 0.4, 0.6, 0.8.


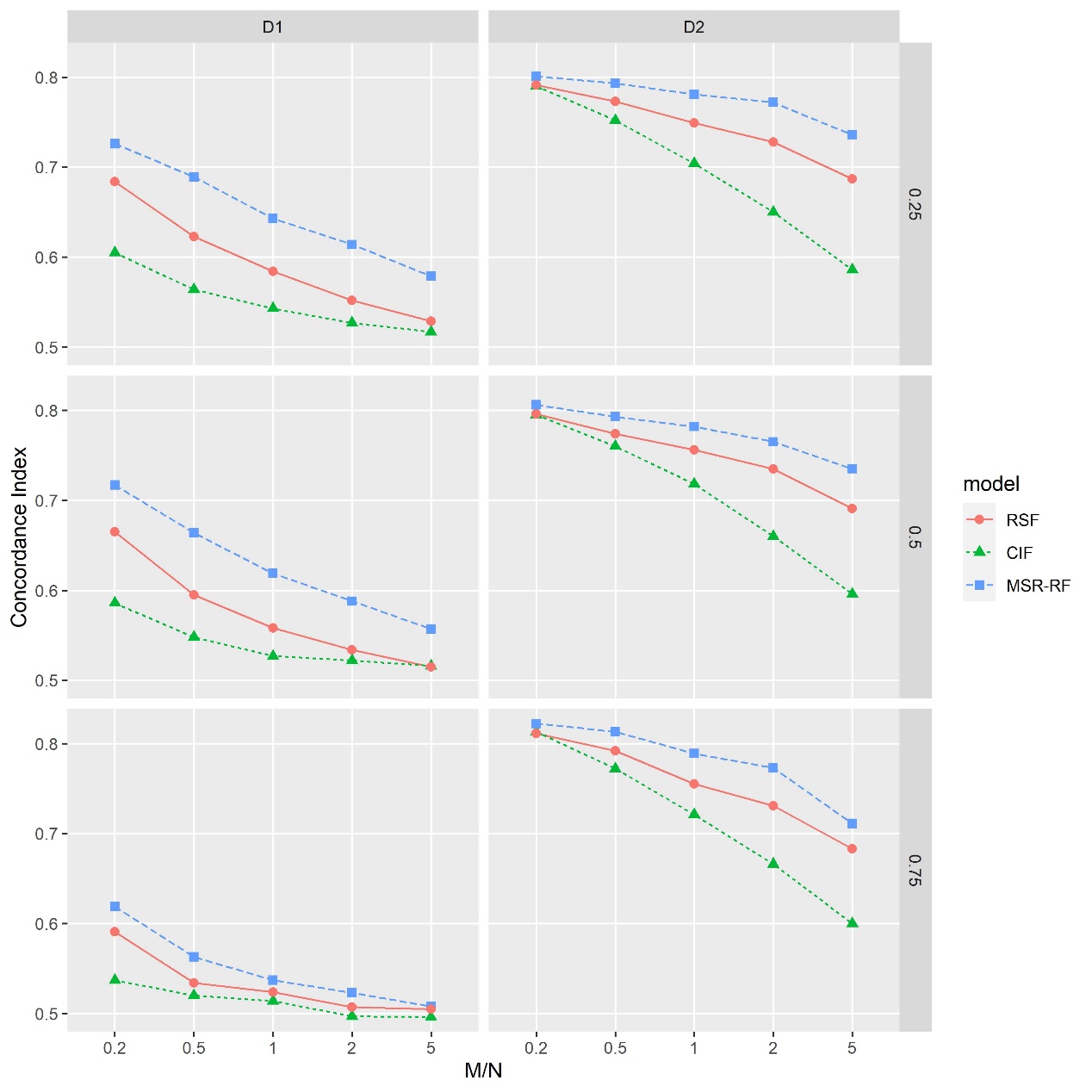


Fig S12 Concordance index for D datasets with RSF, CIF and MSR-RF.

Dataset D was set in a linear form with all variables generated from the standard normal distribution for D1 and the binomial distribution with 0.5 probability for D2. Selection frequency means the rates of the two correct variables $x_{1}$ and $x_{2}$ranking top, whereas $x_{1}$ with coefficient 2 and $x_{2}$ with coefficient 3. The sample size *N* was set to 100; the censoring rate was set to 25%, 50%, 75%. The ratio *M/N*, which means the ratio of the number of covariates *M* to the sample size *N*, was set to 0.2, 0.5, 1, 2, 5.
